# Quercetin enhances survival and axonal regeneration of motoneurons after spinal root avulsion and reimplantation: experiments in a rat model of brachial plexus avulsion

**DOI:** 10.1186/s41232-022-00245-3

**Published:** 2022-12-01

**Authors:** Yanfeng Huang, Xie Zhang, Qionghui Huang, Yaoxing Dou, Chang Qu, Qingqing Xu, Qiuju Yuan, Yan-Fang Xian, Zhi-Xiu Lin

**Affiliations:** 1 School of Chinese Medicine, Faculty of Medicine, The Chinese University of Hong Kong, Shatin, N.T., Hong Kong, SAR China; 2 School of Basic Medical Sciences, Department of Medical Biotechnology, Guangzhou University of Chinese Medicine, Guangzhou, Guangdong People’s Republic of China; 3 The Second Affiliated Hospital of Guangzhou University of Chinese Medicine, Guangzhou, People’s Republic of China; 4 Centre for Regenerative Medicine and Health, Hong Kong Institute of Science & Innovation, Chinese Academy of Sciences, Hong Kong Science Park, Shatin, N.T., Hong Kong, SAR China; 5 Hong Kong Institute of Integrative Medicine, The Chinese University of Hong Kong, Hong Kong, SAR China

**Keywords:** Brachial plexus avulsion, Quercetin, Motoneuron, Oxidative damage, Inflammatory response

## Abstract

**Background:**

Brachial plexus avulsion (BPA) physically involves the detachment of spinal nerve roots themselves and the associated spinal cord segment, leading to permanent paralysis of motor function of the upper limb. Root avulsion induces severe pathological changes, including inflammatory reaction, oxidative damage, and finally massive motoneuron apoptosis. Quercetin (QCN), a polyphenolic flavonoid found in abundance in fruit and vegetables, has been reported to possess anti-oxidative, anti-inflammatory, and neuroprotective effects in many experimental models of both central nervous system (CNS) and peripheral nervous system (PNS) disorders. The purpose of this study was to investigate whether QCN could improve motor function recovery after C5–7 ventral root avulsion and C6 reimplantation in a rat model of BPA.

**Methods:**

The right fifth cervical (C5) to C7 ventral roots were avulsed followed by re-implantation of only C6 to establish the spinal root avulsion plus re-implantation model in rats. After surgery, rats were treated with QCN (25, 50, and 100 mg/kg) by gavage for 2 or 8 consecutive weeks. The effects of QCN were assessed using behavior test (Terzis grooming test, TGT) and histological evaluation. The molecular mechanisms were determined by immunohistochemistry analysis and western blotting.

**Results:**

Our results demonstrated that QCN significantly expedited motor function recovery in the forelimb as shown by the increased Terzis grooming test score, and accelerated motor axon regeneration as evidenced by the ascending number of Fluoro-Ruby-labeled and P75-positive regenerative motoneurons. The raised ChAT-immunopositive and cresyl violet-stained neurons indicated the enhanced survival of motoneurons by QCN administration. Furthermore, QCN treatment markedly alleviated muscle atrophy, restored functional motor endplates in biceps and inhibited the microglial and astroglia activation via modulating Nrf2/HO-1 and neurotrophin/Akt/MAPK signaling pathway.

**Conclusions:**

Taken together, these findings have for the first time unequivocally indicated that QCN has promising potential for further development into a novel therapeutic in conjunction with reimplantation surgery for the treatment of BPA.

**Supplementary Information:**

The online version contains supplementary material available at 10.1186/s41232-022-00245-3.

## Highlights

• Quercetin (QCN) promotes motoneuron survival and accelerates axonal regeneration after brachial plexus root avulsion/re-implantation in rats.

• QCN improves motor function recovery of the forelimb.

• QCN exhibits neuroprotective effects via inhibiting neuroinflammation and upregulating the neurotrophins.

• QCN alleviates the avulsion-induced oxidative damage in rats.

• QCN inhibits the avulsion-induced neuronal apoptosis in rats.

• QCN modulates Nrf2/HO-1 and neurotrophin/Akt/MAPK signaling pathways.

**Supplementary Information:**

The online version contains supplementary material available at 10.1186/s41232-022-00245-3.

## Introduction

Brachial plexus avulsion (BPA), which is a longitudinal spinal cord injury (SCI), involves the tearing off of nerve roots from their pertinent spinal cord segments [1, 2]. It is one of the most severe brachial plexus injuries, mainly as a result of road traffic accidents, sport injuries and difficult deliveries [3, 4]. The short distance between the lesion and the cell body leads to rampant progressive motoneuron death in the early stage of the injury [5]. In addition, the combination of finite inherent axonal regeneration speed and the long distance they have to go through before they can reinnervate their target muscles contribute to chronic distal muscles denervation and eventually permanent motor dysfunction in the upper extremity [6]. About 70% of severe brachial plexus traction injuries clinically involved one or more roots avulsion [7]. Direct surgical reimplantation of the avulsed nerve root to the affected spinal segments can enhance axonal regeneration and reinnervate muscle targets, which is conducive for functional recovery of the hand movement [8, 9]. Nonetheless, simple reimplantation is known to be insufficient to achieve satisfactory outcomes [10].

Spinal root avulsion induces a variety of pathophysiological events involving a wide range of specific genes and proteins associated with inflammation, oxidative stress, and apoptosis, eventually contributing to the death of the affected motoneurons [11, 12]. Primitive pathological changes, such as oxidative damage and inflammatory response, can cause massive neuronal death. After a primary mechanical injury, the secondary cascade generates reactive oxygen species (ROS) that finally results in cell damage and apoptosis [13]. Apoptosis is one of the major pathways that lead to cell death after BPA. Avulsion induced oxidative stress may damage mitochondrial membrane, and result in translocation of B cell lymphoma-2-associated X (Bax) from the cytoplasm to the mitochondria [14]. This process is controlled by B cell lymphoma-2 (Bcl-2) proteins, which either inhibit or promote cell death [15]. Another key factor for BPA is the imbalance of neurotrophic factors, a family of proteins that influence various activities of neuronal cells such as proliferation, growth, differentiation and regeneration [16]. Neurotrophic factors and their receptors are widespread throughout the central and peripheral nervous systems. Neurotrophins such as brain derived neurotrophic factor (BDNF) and nerve growth factor (NGF) activate several apoptosis-related intracellular signaling pathways, including the activation of Akt and mitogen-activated protein kinase (MAPK). BDNF induces signaling through high affinity tropomyosin-related kinase (Trk) [17]. Akt is crucial for cell survival, while MAPK is necessary for neurite outgrowth and maintenance, and both contribute to neuronal plasticity.

**Fig. 1:**
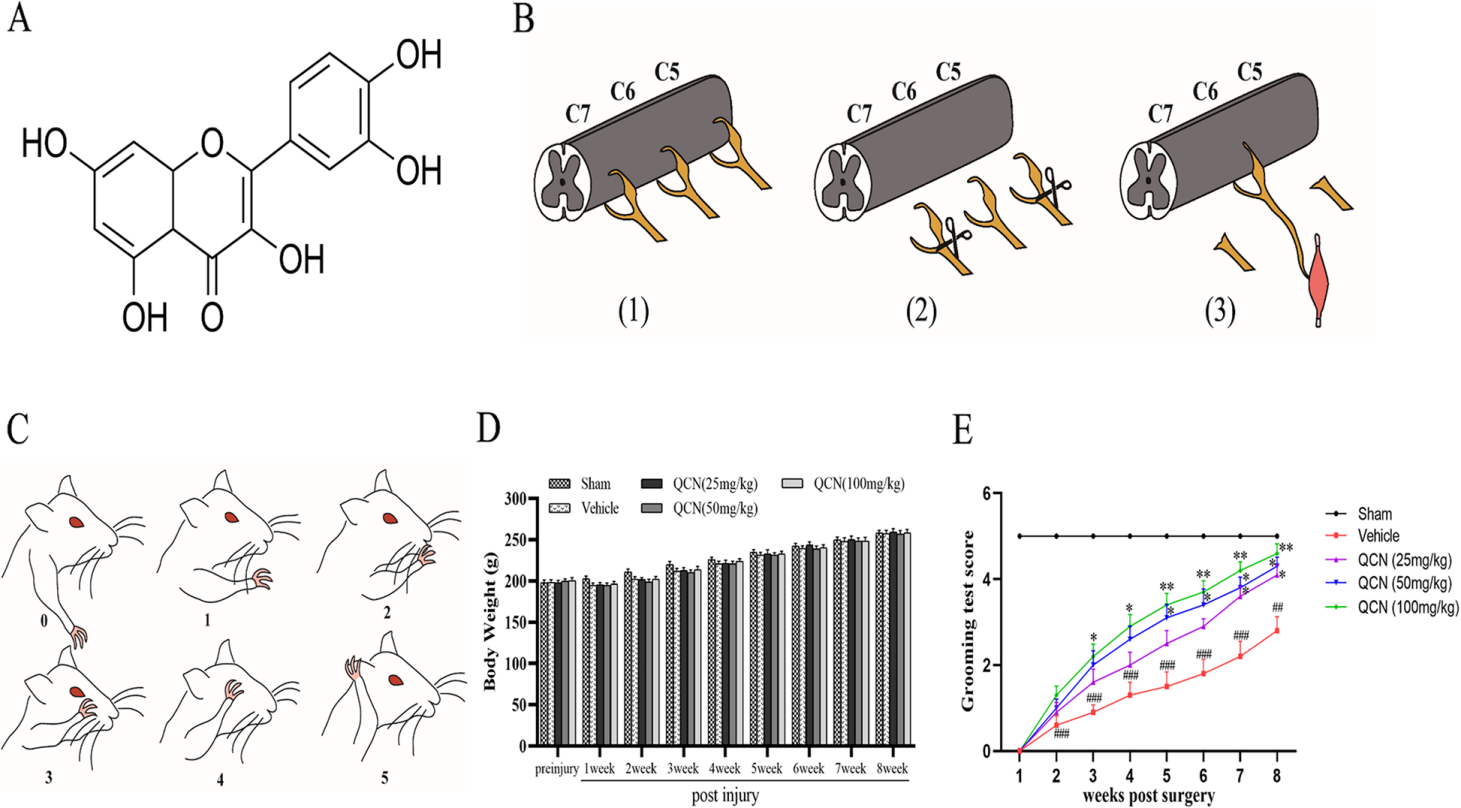
Effects of QCN on the motor function recovery at week 8 post-avulsion. **A** Chemical structure of quercetin (QCN). **B** Schematic diagram showing spinal root avulsion-reimplantation surgical procedures. (1) Normal anatomic construction of C5-C7 spinal cord segments; (2) Avulsion of right C5-7 ventral and dorsal roots; (3) Reimplantation of C6 to the ventral surface of the corresponding spinal segment. **C** Schematic scoring systems of the Terzis grooming test (TGT). **D** The average body weights of rats in different experimental groups. **E** The average TGT scores of rats in different experimental groups. Data are expressed as the mean ± SEM (*n* = 10). ^##^*p* < 0.01 and ^###^ *p* < 0.001 vs the sham group; *p < 0.05 and **p < 0.01 vs the vehicle group

Quercetin (QCN, the chemical structure is shown in Fig. 1A) is a widespread polyphenolic compound found in numerous fruit and vegetables such as onions, broccoli, apples and capers [18, 19]. Studies in rats and pigs have shown that QCN and its metabolites are distributed in various tissues, particularly in the lung, kidney, colon and liver, with lower level found in the brain [20]. QCN exerts protective effects against cardiovascular diseases [21] as well as neurodegenerative diseases [22]. In addition, previous studies have demonstrated that QCN exhibits diverse biological activities, including antioxidant [23], anti-inflammatory [24], and anticancer [25, 26] effects. Moreover, the neuroprotective effect of QCN has been reported in a number of animal models, such as neurodegeneration [27–29], cerebral ischemia [30], traumatic brain injury [31, 32], and spinal cord injury (SCI) [33–35]. It is worth noting that QCN could improve motor function recovery and axonal regeneration, and suppress astrocyte activation in acute SCI animal models [33, 34]. Although the protective effects of QCN against SCI have been reported, the specific mechanisms underlying the therapeutic effect of QCN on BPA remain unexplored. Therefore, in the present study, we aimed to assess the effects of QCN on motor function recovery in a rat model of BPA characterized by spinal ventral root avulsion/reimplantation and illuminate the underlying molecular mechanisms.

## Materials and methods

### Animals

Adult female Sprague–Dawley rats (weighing 180–220 g) were purchased from the Laboratory Animal Services Centre of The Chinese University of Hong Kong (CUHK). The experimental procedures were approved by the Animal Experimentation Ethics Committee, CUHK (No. 18/076/MIS-5-C). All animals were housed under standard environmental conditions at controlled temperature (22 ± 2 °C), humidity (50 ± 10%), and light (12 h light/dark cycle) with free access to standard diet and water.

### Surgical procedures

Spinal root avulsion-reimplantation surgery was performed as per the previously described methods [9, 36], and the detailed procedures were shown in Fig. 1B. For the surgery, the rats were anesthetized by intraperitoneal injection with ketamine (75 mg/kg) and xylazine (10 mg/kg), and then placed in a prone position on a clean surgical table. Under an operative microscope (magnification × 10), the right spine segments from the 4th cervical (C4) to the 2nd thoracic (T2) were carefully exposed and identified based on their location relative to the long spine of T2. Then a dorsal laminectomy was carried out. After opening the dura matter, the right side C5–C7 dorsal and ventral roots were avulsed by traction with a fine glass hook, and parts of C5 and C7 were cut and removed, leaving a crack of about 5 mm between the nerve roots and spinal cord to prevent any reconnection. Meanwhile, the avulsed C6 ventral root was reattached back to the original avulsed site. Extra care was exercised to avoid any injury to the spinal cord. Finally, the skin was stitched and the animals were allowed to recover in individual cages.

### Treatment and grouping

QCN was purchased from Sigma (St. Louis, MO, USA, lot numbers: Q4951, ≥ 98%). Its identity was confirmed by comparing its H^1^ NMR spectra with that published in the literature [37]. Rats were randomly divided into five groups: sham control group, vehicle group, and QCN (25, 50 and 100 mg/kg) groups. Rats were orally administered with QCN (25, 50, and 100 mg/kg), while the rats in the vehicle group received an equal volume of 0.5% carboxymethylcellulose (CMC) for 2 or 8 weeks, respectively. In the sham control group, only the right C5–C7 spinal roots were exposed but not avulsed. It should be noted that the doses of QCN were chosen according to the results of our preliminary study and other investigators [37–39].

### Behavioral test for motor function

After surgery, we performed the upper limbs Terzis grooming test (TGT) once a week to monitor motor function recovery as described by Bertelli and Mira [40]. Briefly, around 3–5 mL water was sprayed on the snout of the experimental rats with a syringe to elicit bilateral grooming behavior of the upper limbs. A 0–5 rating criterion was adopted depending on the highest position where the ipsilateral forelimb could reach. Scoring systems are as follows and depicted in Fig. 1C: grade 0, no response of upper limbs; grade 1, elbow flexion without reaching the nose; grade 2, elbow flexion reaching the snout; grade 3, reaching below the eyes; grade 4, reaching the eyes; grade 5, reaching the ear or beyond.

### Tissue preparation and cresyl violet staining

At the indicated time points, rats were euthanized under anesthesia by intraperitoneal injection of an overdose of pentobarbital sodium and perfused intracardially with 0.9% normal saline, followed by 4% paraformaldehyde (PFA) solution (PBS, pH 7.4). The C6–7 segments of spinal cord and both biceps were harvested, then post-fixed in 4% PFA for 24 h, dehydrated in 30% sucrose for 2–3 days at 4 °C. The C6–7 spinal cord transverse Sects. (25 μm) and biceps (10 μm) were dissected by cryostat (Leica CM1850, Leica Microsystems GmbH, Wetzlar, Germany), and shifted to gelatin-coated slides at − 20 °C before processing. Besides, the biceps were dissected and weighed. Meanwhile, survived neurons in the spinal cord sections were stained with 0.2% (w/v) cresyl violet (Nissl staining) solution according to a previous study [41].

### Retrograde labeling with Fluoro-Ruby (FR)

At 8-week endpoint and four days before perfusion, 4 rats from each group were selected to perform retrograde labeling study using Fluoro-Ruby (FR). The rats were anesthetized with ketamine (80 mg/kg) and xylazine (8 mg/kg) and the ipsilateral musculocutaneous nerve was exposed and identified. A total of 2 μL of the FR (dextran tetramethylrhodamine, 10,000 MW, Thermo Fisher Scientific, 10% in sterilized water) was slowly injected into the ipsilateral musculocutaneous nerve using a fine glass micropipette and the injection position was gripped using a microsurgery forceps for approximately 10 s to avoid any leakage. Then the skin was stitched and the animals were returned to their cages. These animals were allowed to survive for an additional 4 days to permit tracer transport, then perfused and fixed with 4% PFA solution, and the C6–7 spinal cords were harvested and sliced (25 μm). Slices were observed under a fluorescence microscope with a 580-nm filter and the number of retrogradely labeled cells was determined on every other sections. The averaged numbers of the labeled cells at each group were calculated.

### Immunohistochemistry

After blocking with 5% bovine serum albumin (BSA) for 1 h, the spinal cord tissue slices were incubated overnight at room temperature (RT) with different primary antibodies: goat anti-choline acetyltransferase (ChAT; 1:1000; Millipore), rabbit anti-ionized calcium binding adaptor molecule 1 (Iba1; 1:1000, Wako), mouse anti-glial fibrillary acidic protein (GFAP; 1:1000, Sigma), mouse anti-neuronal nitric oxide synthases (nNOS; 1:1000, Invitrogen), and rabbit anti-low-affinity nerve growth factor receptor (p75; 1:1000, Millipore). After repeated washes in 0.01 M phosphate-buffered saline (PBS, pH 7.4), slices were incubated with corresponding secondary antibodies conjugated with Alexa Fluor-488 or 594 (1:1000, Invitrogen) for 4 h at room temperature in the dark. Besides, sections of biceps were stained with α-Bungarotoxin (α-BTX) antibody (1:1000; Alexa Fluor 488 conjugated; Invitrogen) for 2 h at room temperature in the dark. Following thorough washes, fluorescence mounting medium (Dako, Copenhagen, Denmark) was used to mount tissue onto coverslips for microscopic examination. Finally, fluorescent images were captured using a Zeiss fluorescence microscope (Zeiss, Gottingen, Germany) equipped with an ORCA-Flash 4.0 v2 digital CMOS camera (Hamamatsu Photonics, Iwata City, Japan).

### Cell counting

Data for each parameter was obtained from 10 to 15 sections of spinal segments from each animal and analyzed using ImageJ software (version 1.8.0, National Institutes of Health, USA). The survival rate of ChAT-stained motoneurons of the spinal segments was counted as the percentage of neurons in the ipsilateral ventral horn to those in the contralateral side as described previously [41]. The number of nNOS positive cells was counted as the mean value of neurons in each rat [42]. The mean area of GFAP-positive microglia and Iba1-positive astrocytes in each limiting square was quantified according to the method described previously [43]. About 100–200 motor endplates from each musculus biceps brachii of each animal were randomly chosen to photograph. Average motor endplate area from six rats was calculated in each group using ImageJ software [44].

### Western blotting analysis

C6–7 ventral spinal cord tissues were disrupted by homogenization in RIPA buffer containing 1% protease inhibitor cocktail. After centrifugation (12,000 rpm, 15 min) at 4 °C, the supernatants were collected. Protein concentrations were determined using the BCA protein assay kit (Thermo Fisher Scientific, USA). The protein lysates were separated by 10% SDS-PAGE and electrophoretically transferred onto PVDF membranes (Roche Applied Science, Germany). After blocking with 5% nonfat milk in TBS-T for 1 h, membranes were incubated with rabbit anti-nuclear factor-erythroid 2-related factor 2 (Nrf2, 1:1000, Abcam), rabbit anti-heme oxygenase-1 (HO-1, 1:1000, Abcam), rabbit anti-nerve growth factor (NGF, 1:1000, Cell Signaling Technology), rabbit anti-brain-derived neurotrophic factor (BDNF, 1:1000, Santa Cruz), rabbit anti-phospho-Akt (p-Akt, 1:1000, Cell Signaling Technology), rabbit anti-Akt (1:1000, Cell Signaling Technology), rabbit anti-B cell lymphoma-2 (Bcl-2, 1:1000, Cell Signaling Technology), mouse anti-B Cell lymphoma-extra large (Bcl-xL, 1:500, Santa Cruz), rabbit anti-Bcl-2-associated X (Bax, 1:1000, Cell Signaling Technology), mouse anti-Caspase 9 (1:500, Santa Cruz); mouse anti-Caspase 3 (1:500, Santa Cruz), mouse anti-B-Raf (1:500, Santa Cruz), rabbit anti-phospho-extracellular signal-regulated kinase (p-ERK, 1:1000, Cell Signaling Technology), rabbit anti-ERK (1:1000, Cell Signaling Technology), mouse anti-phospho-MAPK/ERK kinase (p-MEK, 1:500, Santa Cruz), mouse anti-MEK (1:500, Santa Cruz), and rabbit anti-GAPDH (1:10,000, Abcam) antibodies overnight at 4 °C, and then incubated with secondary antibodies for 1 h at room temperature. The protein bands were detected using an enhanced chemiluminescence (ECL) substrate reagent kit (Invitrogen, USA) and quantified by ImageJ software using GAPDH as the internal control.

### Statistical analysis

Data were expressed as the mean ± standard error mean (SEM). Statistical analysis was carried out using one-way analysis of variance (ANOVA), followed by Dunnett’s test with SPSS 26.0 software (IBM, New York, USA). The *p* < 0.05 was considered statistically significant.

## Results

### Effects of QCN on the body weight and motor function recovery of BPA rats

As shown in Fig. 1D, body weight in all surgical groups decreased at 1 week postoperatively as compared to that of the Sham group, although the difference was only marginal (*p* > 0.05). From week 2 to week 8 after surgery, the body weight of rats in all experimental groups gradually increased. Besides, no significant differences in body weight were found among vehicle and QCN-treated groups (*p* > 0.05), suggesting that QCN exerted no obvious adverse effect during the experimental period.

The motor function recovery of the upper right limb in rats was assessed by TGT test weekly. Before avulsion surgery, all of the animals displayed normal movement of the right forelimb, with a mean TGT score of 5. However, all animals in the avulsion-injured groups had a TGT score of 0 at the first week of post-injury, indicating a loss of total motor function and confirming a successful surgical operation. Functional recovery started from week 2 after surgery in both of the QCN and vehicle treated groups, and the TGT scores increased every week in all of the avulsion-injured groups (Fig. 1E). Moreover, the averaged TGT scores from week 5 to week 8 after surgery in the QCN-treated groups (50 and 100 mg/kg) were markedly improved (*p* < 0.05 and *p* < 0.01, respectively, for all time points), when compared with the vehicle-treated group. Significant differences in the averaged TGT scores were detected between the low dose of QCN (25 mg/kg) and the vehicle-treated group at week 7 post-surgery (*p* < 0.05). Taking together, the combined treatment of QCN and reimplantation was able to achieve a better functional recovery.

### Effects of QCN on the regeneration of motoneurons of BPA rats

**Fig. 2:**
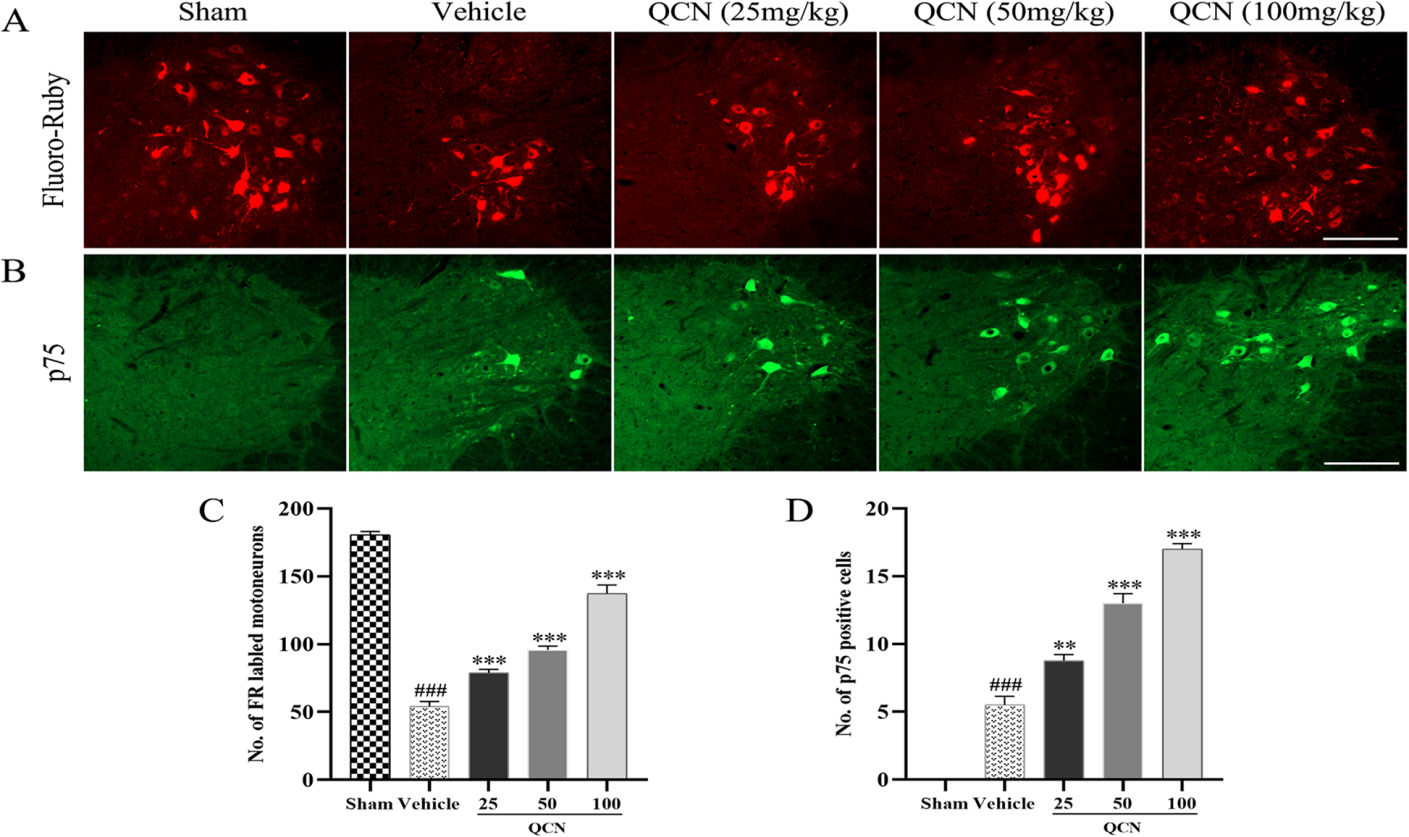
Effects of QCN on the regeneration of motoneurons of BPA rats at week 8 post-avulsion. Representative images of **A** Fluoro-Ruby (FR) labeled motoneurons, and **B** P75-positive motoneurons in the ipsilateral C6–7 ventral spinal segments. Quantification of **C** FG-labeled cells and **D** P75-immunoreactive (IR) cells of each group at 8 weeks. Data are expressed as the mean ± SEM (*n* = 4). ^###^ *p* < 0.001 vs the sham group; **p < 0.01 and ***p < 0.001 vs the vehicle group. Scale bar 200 μm

To further explore whether the functional enhancements were associated with more axonal regeneration of motoneurons, we first performed retrograde tracing by injecting Fluoro-Ruby (FR) into the distal part of musculocutaneous nerve, followed by quantifying FR labeled neurons in the ventral horn of spinal cord. As observed at week 8 after surgery (Fig. 2A, C), a dramatic decrease was found in the number of FR-labeled cells in the rats treated with the vehicle as compared to the sham group (*p* < 0.001). However, the number of FR-labeled cells on the lesional side were statistically increased (*p* < 0.001 for all) after treatment with QCN (25, 50, and 100 mg/kg), as compared with the vehicle group. The results suggest that the QCN-treated animals have higher percentage of surviving motoneurons which have regenerated their axons into the re-implanted root.

Moreover, it has been reported that p75 is associated with motoneuron survival and axonal regeneration, and all of the regenerated motoneurons re-expressed p75 in this animal model at week 8 after surgery [45]. Hence, we performed immunohistochemistry on p75 to quantitatively measure the successful regeneration of axons of the spinal motoneurons. As shown in Fig. 2B, D, no p75 signal could be found in the motoneurons in the sham control group. Expression of p75 in the ventral horn of the spinal segments was sharply upregulated in all groups after root avulsion and reimplantation surgery at week 8 when compared with those in the sham group. Besides, a marked increase in the quantity of the p75 positive motoneurons was detected in the animals treated with QCN (25, 50, and 100 mg/kg) (*p* < 0.01, *p* < 0.001, and *p* < 0.001, respectively), when compared to those in the vehicle group. The results were congruent with that of FR labeling. All these experimental findings amply indicate that QCN and reimplantation collectively improved axonal regeneration of the avulsed motoneurons.

### Effects of QCN on survival of injured motoneurons of BPA rats

**Fig. 3:**
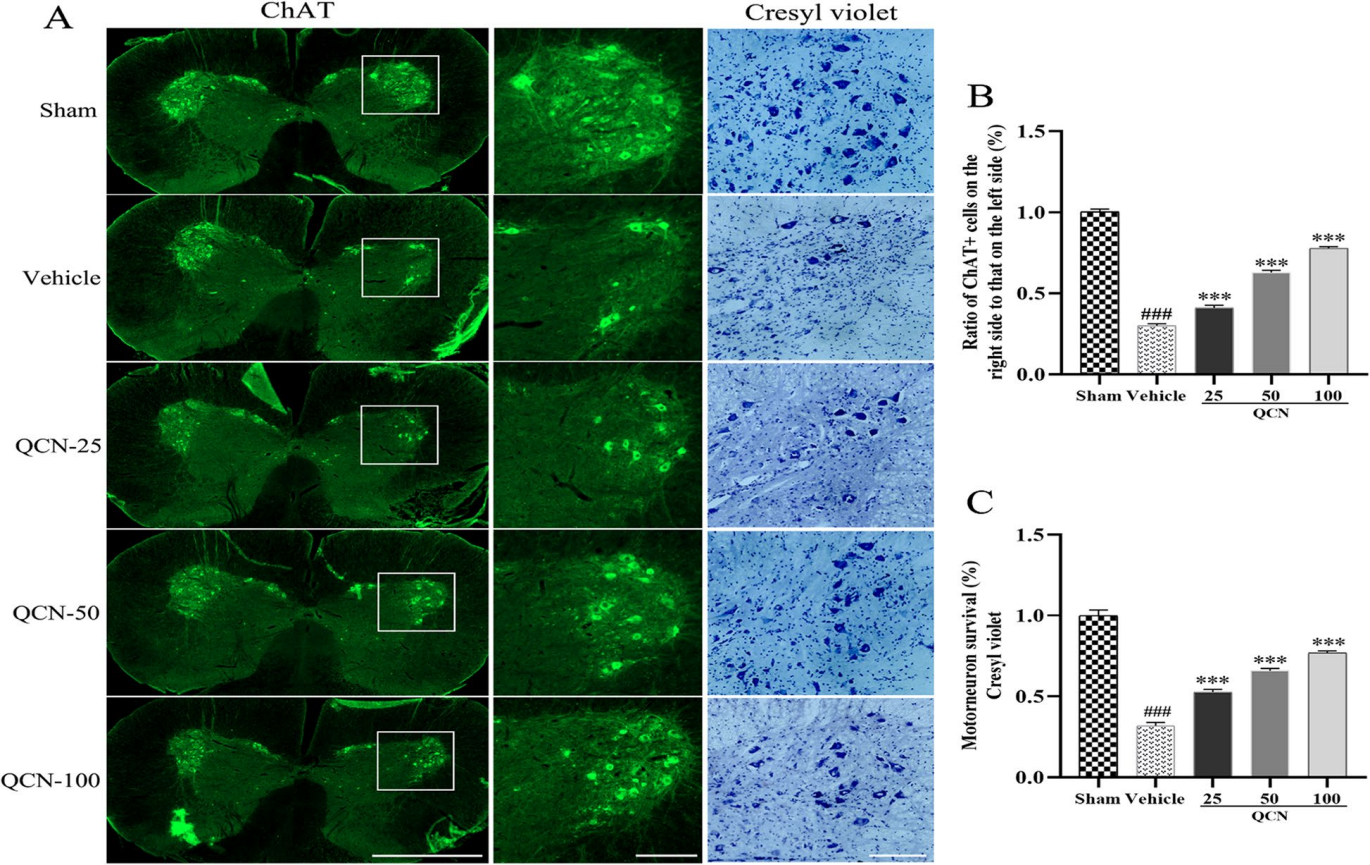
Effects of QCN on survival of injured motoneurons of the BPA rats at week 8 post-avulsion. Representative images of **A** ChAT and cresyl violet staining motoneurons in the ipsilateral C6–7 ventral spinal segments. The survival rate of **B** ChAT-positive motoneurons and **C** Cresyl violet stained motoneurons of each group in the ipsilateral to contralateral ventral spinal segments at week 8. Data are expressed as the mean ± SEM (*n* = 6). ^###^ *p* < 0.001 vs the sham group; ***p < 0.001 vs the vehicle group. Scale bar 100 μm for ChAT on the left; 200 μm for ChAT on the right; and 200 μm for cresyl violet

Since it is vital to maintain the survival of the avulsed spinal motoneurons to achieve functional recovery, we examined whether QCN could enhance the survival rate of motoneurons at week 8 after the ventral root avulsion and reimplantation surgery. Cresyl violet staining for the Nissl substance and ChAT (a marker for motoneurons) immunohistochemical staining are commonly used to evaluate survival of the injured motoneurons in the long-term avulsion injury experimental models as described in a previous study [46, 47]. The survival rate of motoneurons was estimated as the ratio of ipsilateral/contralateral motoneurons in the ventral horn of spinal cord. No statistical differences were found in the number of motoneurons on the contralateral side in all groups (data not shown). As displayed in Fig. 3, at week 8 post-surgery, both cresyl violet staining and ChAT immunostaining showed that root avulsion resulted in less motoneurons in the ventral horns of lesional sides (*p* < 0.001 for all), when compared to the intact motoneurons in the sham group. On the contrary, a higher survival rate of motoneurons was detected in the QCN (25, 50, and 100 mg/kg)-treated animals as shown by cresyl violet staining and ChAT immunostaining (*p* < 0.001 for all), when compared with that in the vehicle rats. Generally, these results indicate that QCN effectively mitigated the motoneuron death induced by mechanical avulsion injury.

### Effects of QCN on muscle atrophy of BPA rats

**Fig. 4:**
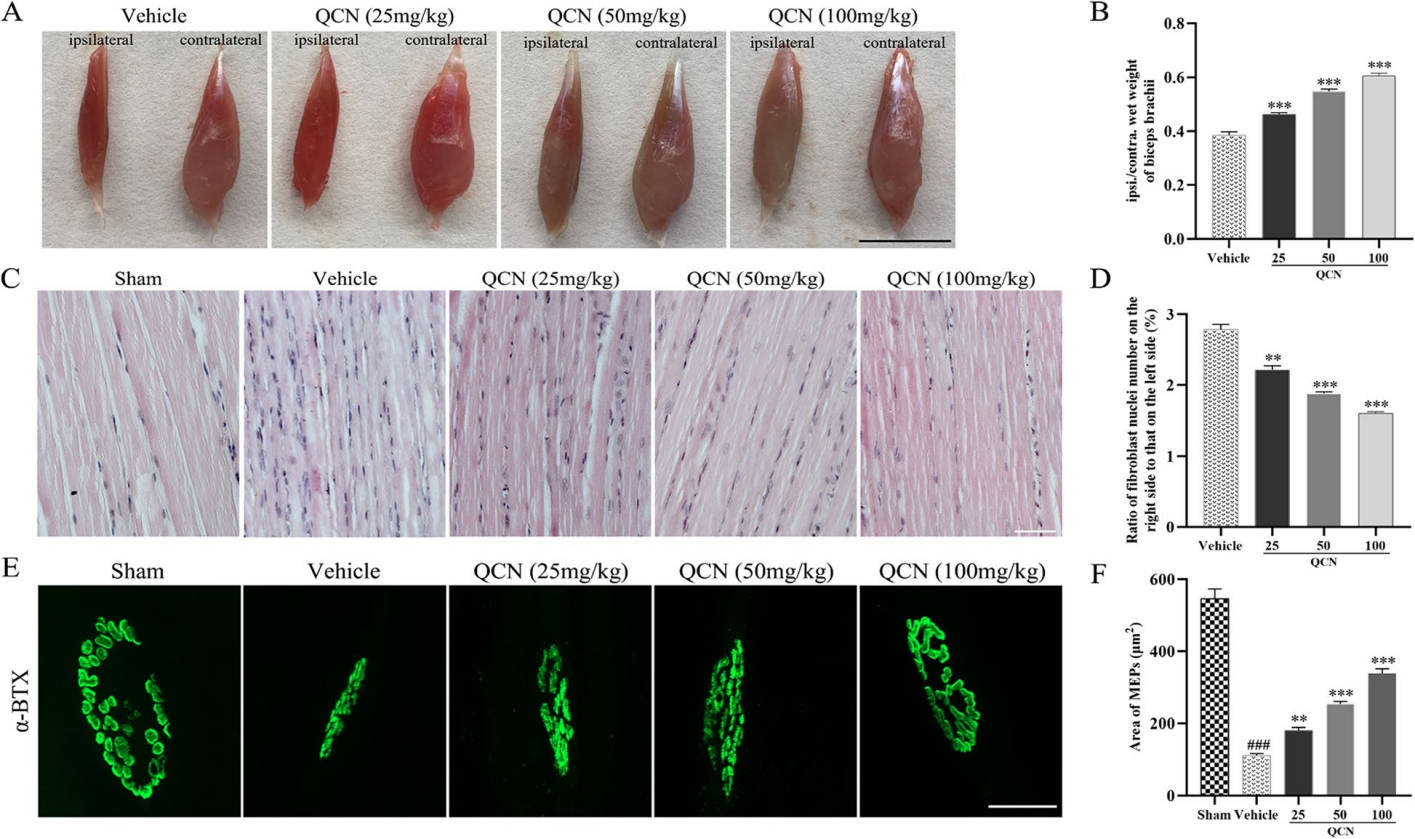
Effects of QCN on muscle atrophy of the BPA rats at week 8 post-avulsion. **A** Representative images of biceps muscles in both of the ipsilateral and contralateral sides. **B** The rate of wet weight of the biceps muscles. **C** Histological images of longitudinal bicep sections with H & E staining. **D** The percentage of fibroblast nuclei number in the ipsilateral to contralateral biceps. **E** Representative Z-Stack images of biceps muscles sections stained with FITC-conjugated α-BTX. **F** Average area of MEPs in each group. Data are expressed as the mean ± SEM (*n* = 6). ^###^ *p* < 0.001 vs the sham group; **p < 0.01 and ***p < 0.001 vs the vehicle group. Scale bar 1 cm in A, 50 μm in **C**, 20 μm in **E**

To investigate whether QCN remitted the degree of muscle atrophy after spinal root avulsion and reimplantation surgery, we weighed the biceps brachii muscles of both ipsilateral and contralateral forelimbs at week 8 after surgery, and the ratio of the wet weight of the biceps brachii muscle on the ipsilateral side to that on the contralateral side was calculated. As shown in Fig. 4A, B, all surgical groups exhibited a dramatic drop in the wet weight of biceps of the lesional side when compared to the contralateral side. However, less muscle weight loss (*p* < 0.001 for all) was found in the animals treated with QCN (25, 50, and 100 mg/kg), when compared with those in the vehicle-treated animals.

We also evaluated the pathological alterations of biceps by hematoxylin–eosin (H & E) staining at week 8 after surgery. As revealed in Fig. 4C, D, the muscle fibers in the vehicle group displayed shrunken sarcoplasm and higher amounts of fibroblasts (*p* < 0.001), when compared to those in the sham group, suggesting severe muscular atrophy. In contrast, muscle fibers were bigger and with clear myocyte nuclei and less extensive fibrosis in the rats treated with QCN (25, 50, and 100 mg/kg) (*p* < 0.01, *p* < 0.001, and *p* < 0.001, respectively). Taken together, these findings demonstrated that QCN could effectively ameliorate muscle atrophy of the BPA rats.

### Effects of QCN on motor endplates of BPA rats

Motor endplates (MEPs) are the postsynaptic folds of neuromuscular junctions and contain closely clumped acetylcholine receptors (AChRs), which play a vital role in regulating contractile activity of skeletal muscles [48]. Alpha-bungarotoxin (α-BTX) can specifically bind with AChRs and is widely used to stain muscle sections for examining the reservation of MEPs after BPA. In the vehicle-treated rats with reimplantation alone, MEPs tended to be smaller and faintly stained with an ambiguous appearance at week 8 after surgery (*p* < 0.001), when compared with the biceps in the sham group (Fig. 4E), suggesting significant loss of AChRs as a result of unsubstantial reinnervation. On the other hand, motor endplates in the QCN (25, 50, and 100 mg/kg) groups were larger in size and clearer in appearance (*p* < 0.01, *p* < 0.001, and *p* < 0.001, respectively) than those in the vehicle group (Fig. 4F). These data indicate that combination of QCN and reimplantation expedited motor axon regeneration and enabled reaching of the innervated target muscle more efficiently, thereby facilitating motor function recovery.

### Suppressive effects of QCN on activation of microglia and astrocytes in the BPA rats

**Fig. 5:**
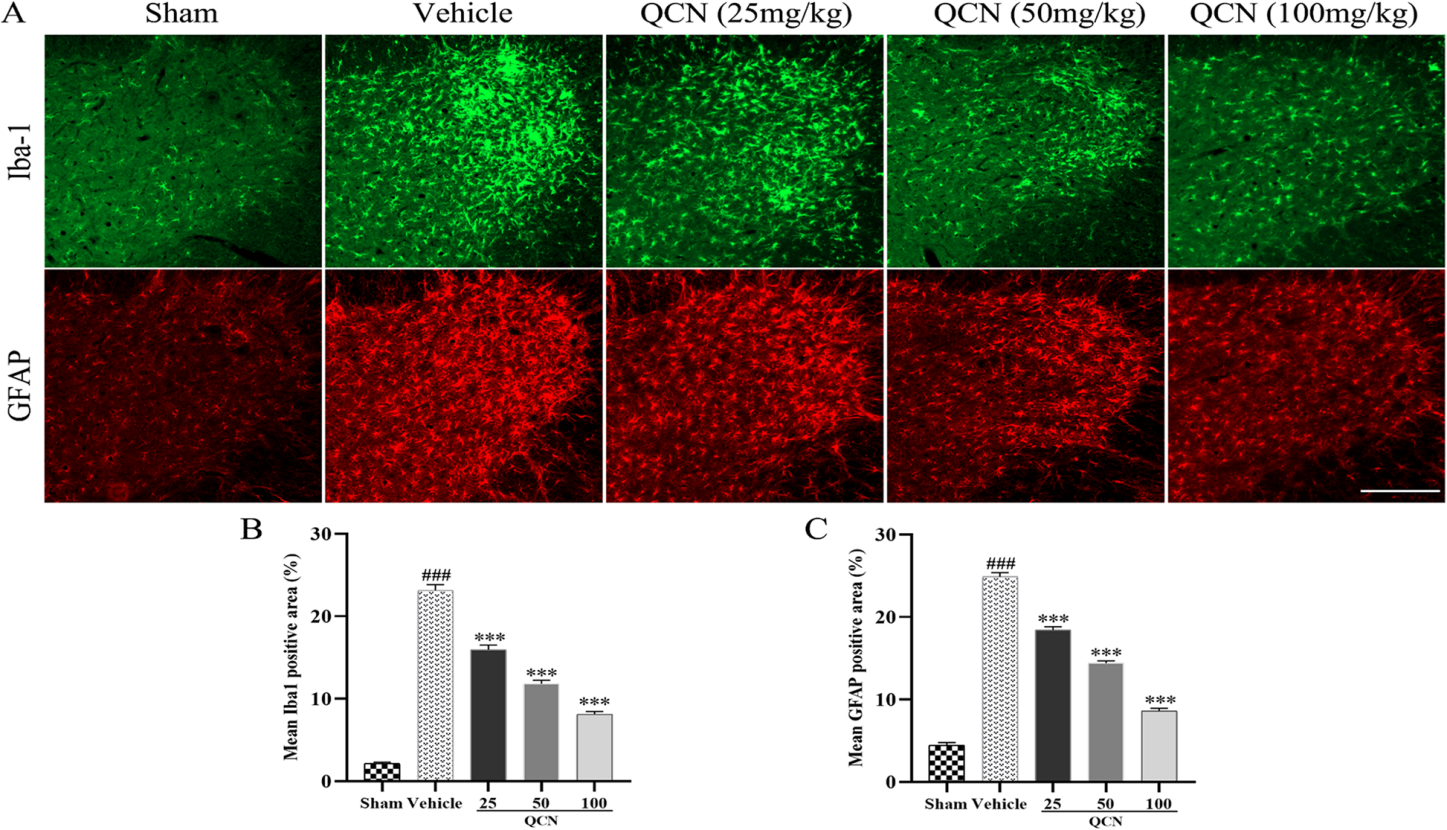
Inhibitory effects of QCN on the activation of microglia and astrocytes of the BPA rats at week 8 post-avulsion. **A** Representative images of Iba1 (green) and GFAP (red) staining cells in the ipsilateral C6–7 ventral spinal segments at 8 weeks post-avulsion injury. Mean **B** Iba1-immunoreactive and **C** GFAP-immunopositive area in the ipsilateral C6–7 ventral spinal segments from all groups. Data are expressed as the mean ± SEM (*n* = 6). ^###^ *p* < 0.001 vs the sham group; ***p < 0.001 vs the vehicle group. Scale bar 200 μm

To determine the effects of QCN on microglia and astrocyte activation after avulsion injury, we detected the Iba1-positive cells (microglia) and GFAP-positive cells (astrocytes) by performing immunohistofluorescence in the spinal cord of the ventral horn at week 8 after post-avulsion. As depicted in Fig. 5, when compared with the sham group, sharp increases in both microglia (*p* < 0.001) and astrocytes (*p* < 0.001) were observed in the vehicle group by fluorescence microscopy, indicating that avulsion injury elicited neuroinflammatory reaction in the lesional site of the spinal cord. While treatment with QCN (25, 50, and 100 mg/kg) significantly suppressed the microglia activation (*p* < 0.001 for all) and the generation of reactive astrocytes (*p* < 0.001 for all) at the corresponding spinal ventral horn, as compared with the vehicle-treated spinal cords. The above results amply demonstrated that QCN could inhibit the avulsion-induced neuroinflammatory infiltration through suppressing the activation of glial cells.

### Effects of QCN on oxidative stress of BPA rats

**Fig. 6:**
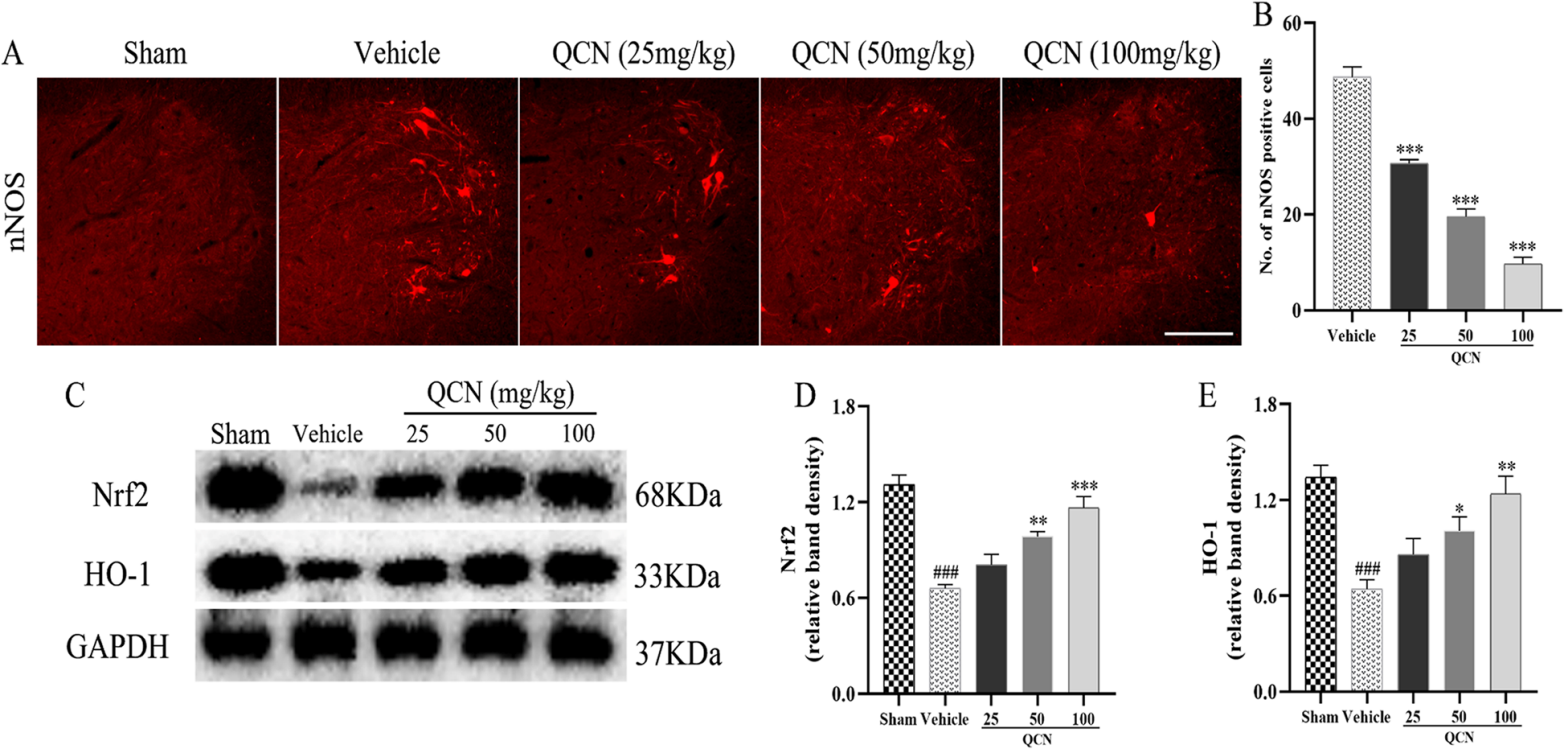
Effects of QCN on oxidative stress of the BPA rats at week 2 post-avulsion*.* **A** Representative images of nNOS staining cells in the ipsilateral C6–7 ventral spinal segments at 2 weeks post-avulsion injury. **B** Quantification of nNOS staining cells of each group. (Scale bar 200 μm. *n* = 6). **C** The protein levels of Nrf2 and HO-1 as determined by western blot. Quantification of **D** Nrf2 and **E** HO-1 protein in the ventral horn of the C6–7 spinal segments. Data are expressed as the mean ± SEM (*n* = 3). ^###^ *p* < 0.001 vs the sham group; *p < 0.05, **p < 0.01, and ***p < 0.001 vs the vehicle group

Previous studies have shown that an excess of oxidative stress gives rise to severe motoneuron death in an avulsion injury [49–51]. nNOS is an enzyme used extensively as a biochemical marker of oxidative stress in spinal motoneurons and is regarded as a signal of the imminent death of the injured cells [52]. As illustrated in Fig. 6A, B, no nNOS signals were found in the spinal ventral horns of the sham-operated rats. In the vehicle group rats, however, there were numerous nNOS positive neurons in the ipsilateral sides of the ventral horns at 2 weeks after root-avulsion. However, in the QCN groups, a significant decrease was observed in the number of nNOS-immunoreactive neurons in the dorsal horn ipsilateral side to the avulsion (*p* < 0.001).

Evolving evidence has manifested that the activation of Nrf2/HO-1 pathway is implicated in the protection against spinal cord oxidative injury [53, 54]. We further examined the effect of QCN on the regulation of Nrf2 and HO-1 expression under oxidative stress at week 2 post-avulsion. Western blot data showed that the Nrf2 and HO-1 expression decreased markedly (*p* < 0.001 for both) 2 weeks after avulsion in the vehicle group, as compared with the sham control (Fig. 6C–E). In contrast, rats in the two QCN-treated groups (50 and 100 mg/kg) showed a significant increase in Nrf2 (*p* < 0.01 and *p* < 0.001, respectively) and HO-1 levels (*p* < 0.05 and *p* < 0.01, respectively), as compared to those in the vehicle control group (Fig. 6D, F). These results clearly indicate that QCN was capable of mitigating the avulsion-induced oxidative stress.

### Effects of QCN on neurotrophins in the spinal cord of BPA rats

**Fig. 7:**
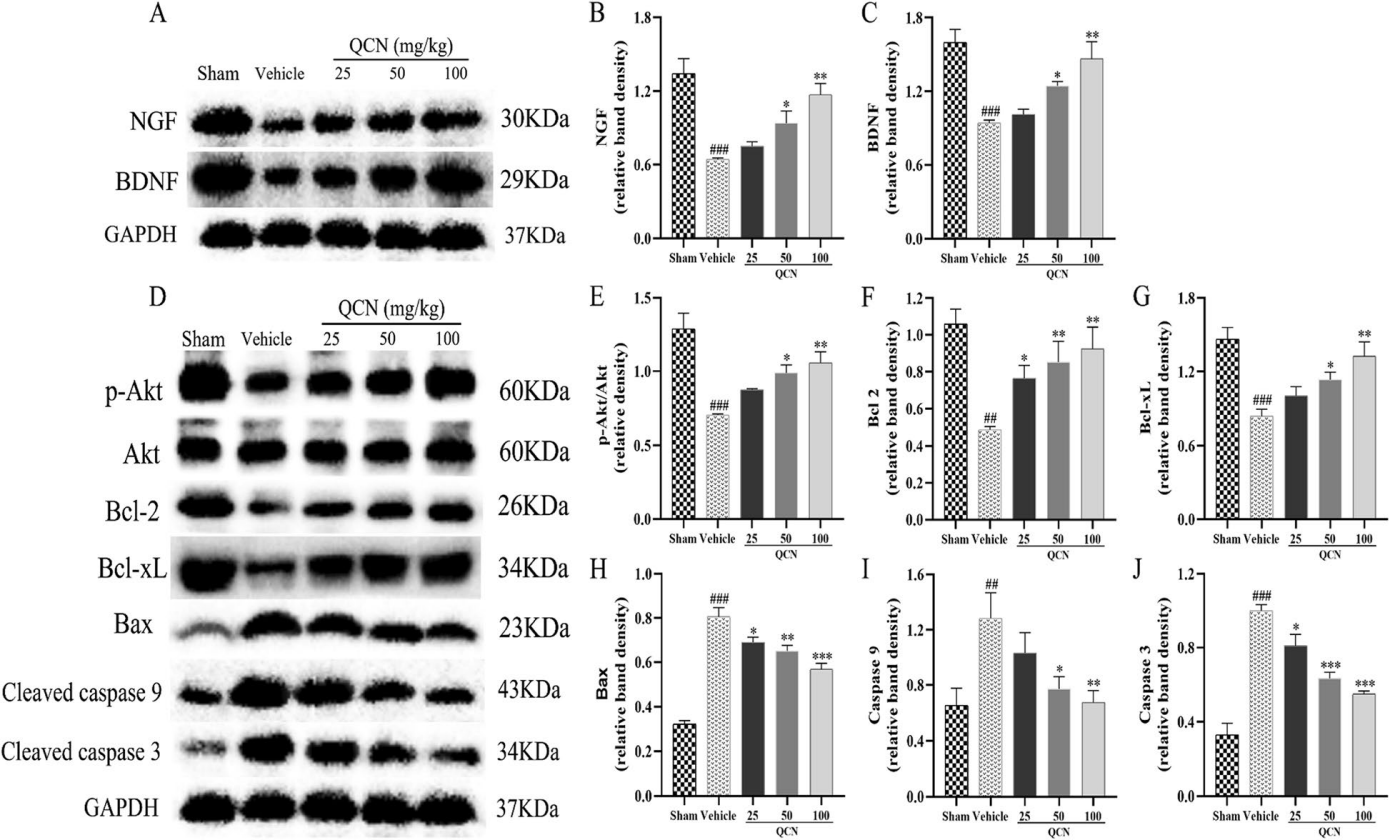
Effects of QCN on neurotrophins and Akt signaling pathway in the spinal cord of the BPA rats at week 8 post-avulsion. **A** The protein levels of BDNF and NGF as determined by western blot. Quantification of **B** BDNF and **C** NGF protein in the ventral horn of the C6–7 spinal segments. **D** The protein levels of p-Akt, Bcl-2, Bcl-xL, Bax, Caspase-9, and Caspase-3 as determined by western blot. Quantification of **E** p-Akt/Akt, **F** Bcl-2, **G** Bcl-xL, **H** Bax, **I** cleaved caspase-9, and **J** cleaved caspase-3 proteins in the ventral horn of the C6–7 spinal segments. Data are expressed as the mean ± SEM (*n* = 3). ^###^ *p* < 0.001 vs the sham group; *p < 0.05, **p < 0.01, and ***p < 0.001 vs the vehicle group

Neurotrophins are known to be promoters of neuronal survival, and regulate many aspects of neuronal development and function, including synapse formation and synaptic plasticity [17, 55, 56]. BDNF and NGF protein expression levels were quantified in the spinal cord of BPA rats by western blotting at week 8 after the root-avulsion (Fig. 7A–C). Densitometric analysis of the protein bands showed a significant reduction in the expression levels of BDNF (*p* < 0.001) and NGF (*p* < 0.001) in the vehicle group rats, as compared to the sham control group (Fig. 7B, C). However, the administration of medium and high doses of QCN treatment (50 and 100 mg/kg) to BPA rats caused a significant increase in the levels of BDNF (*p* < 0.05 for both) and NGF (*p* < 0.05 for both) in the ventral horn of C6–7 spinal cord sections, as compared to the vehicle group.

### Effects of QCN on apoptotic markers and Akt signaling pathway in the spinal cords of BPA rats

To reveal the effect of QCN on apoptosis-associated proteins in the spinal cords after brachial plexus injury, the expression of Akt signaling pathway was assessed at week 8 following BPA surgery. As illustrated in Fig. 7D–J, the protein expressions of Bcl-2 (*p* < 0.01) and Bcl-xL (*p* < 0.001), and the ratio of p-Akt/Akt (*p* < 0.001) in the ventral horn of the spinal segments were significantly lower (*p* < 0.01 for all) (Fig. 7E–G), while the expressions of Bax (*p* < 0.001), cleaved caspase-9 (*p* < 0.01) and cleaved caspase-3 (*p* < 0.001) were significantly higher (Fig. 7H–J) in the rats of the vehicle group, as compared to the sham control group. However, the administration of medium and high doses of QCN (50 and 100 mg/kg) markedly accentuated the expressions of Bcl-2 (*p* < 0.05 for both) and Bcl-xL (*p* < 0.05 and *p* < 0.01), and the ratio of p-Akt/Akt (*p* < 0.05 and *p* < 0.01), while attenuated the levels of Bax (*p* < 0.01 and *p* < 0.001), Caspase-9 (*p* < 0.05 and *p* < 0.01), and Caspase-3 (*p* < 0.001 for both) in the spinal cords of BPA rats, when compared with the vehicle group.

### Effects of QCN on MAPK signaling pathway in the spinal cords of BPA rats

**Fig. 8:**
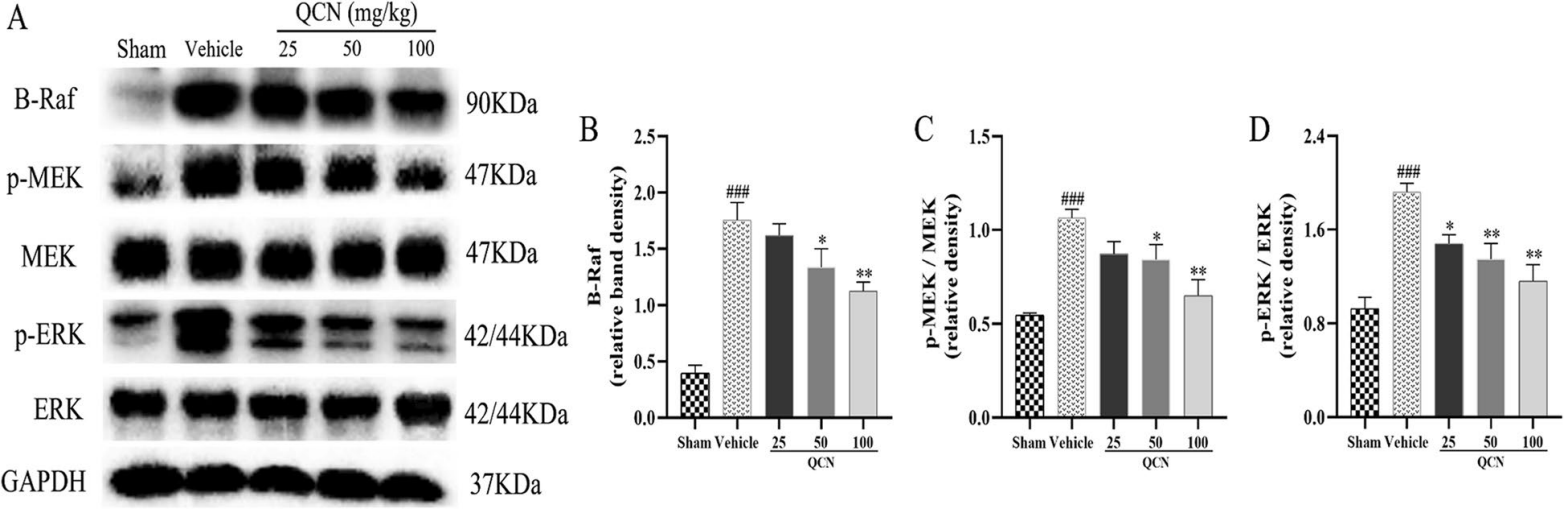
Effects of QCN on MAPK signaling pathway in the spinal cords of BPA rats at week 8 post-avulsion. **A** The protein levels of B-Raf, p-MEK and p-ERK as determined by western blot. Quantification of **B** B-Raf, **C** p-MEK to MEK, and **D** p-ERK to ERK proteins in the ventral horn of the C6-7 spinal segments. Data are expressed as the mean ± SEM (*n* = 3). ^###^ *p* < 0.001 vs the sham group; *p < 0.05 and **p < 0.01 vs the vehicle group

MAPK pathway plays diverse roles in neuronal development and survival. It regulates neuronal differentiation including neurite outgrowth and maintenance [57]. Our western blot results showed that, at week 8 after surgery, the protein expression of B-Raf (*p* < 0.001), the ratios of p-MEK/MEK (*p* < 0.001) and p-ERK/ERK (*p* < 0.001) were significantly increased in the vehicle group, as compared with the sham control group. QCN (50 and 100 mg/kg) significantly decreased the relative level of B-Raf (*p* < 0.05 and *p* < 0.01), the ratios of p-MEK/MEK (*p* < 0.05 and *p* < 0.01) and p-ERK/ERK (*p* < 0.01 for both) in the spinal cords of BPA rats, as compared with the vehicle group (Fig. 8). These results unambiguously indicate that QCN is able to regulate the protein expressions of MAPK pathway-related molecules in the ventral spinal cords of BPA rats, thereby conferring protection to the neurons upon avulsion injury.

## Discussion

Spinal root avulsion in murine is one of the most widely used animal models for BPA research. Its various characteristics, including massive death of motoneurons, intensive degeneration of axons and progressive atrophy of muscles, are similar to human BPA [2]. For these reasons, this experimental model has been regarded as a reliable and reproducible model to provide a good opportunity to study the phenomena of neuronal death and survival after spinal cord injuries and their underlying mechanisms [52]. In addition, surgical reimplantation of avulsed ventral roots has been confirmed to be valid in extricating injured motoneurons, enhancing axon regeneration, and even facilitating functional reinnervation of peripheral targets [58–60]. Nonetheless, reimplantation alone is not enough to achieve satisfactory reinnervation experimentally and clinically [10]. Combination of multiple treatment regimens is vital to promote functional recovery from BPA injury after nerve reimplantation [61]. In our well-established animal model, we intentionally did not re-implant the C5 and C7 roots to minimize the amount of potential axonal sprouting from the spared fibers. Thus, most of the muscle reinnervation was forced to come from re-implanted C6 [36].

As mentioned previously, the flavonoid QCN has been shown to improve the outcome of motor function recovery and increase neuron survival, as well as protect neurons after SCI [33, 34]. Functional recovery and neuronal survival are the fundamental aim in all the therapies for root avulsion injury [62]. Therefore, we first perform TGT of the upper limb to evaluate whether QCN enabled better motor function recovery after spinal root avulsion injury/reimplantation surgery. Then, we used the cresyl violet staining and motoneuron marker ChAT to assess the survival of the affected motor neurons which has been confirmed to be of ultimate importance for axonal regeneration [41]. Moreover, a number of previous studies, which showed no significant difference in motoneuron counts between ChAT and cresyl violet staining, support our results [63]. In our study, the reduction of TGT scores and loss of motoneurons were effectively recovered in the rats of the QCN groups.

FR (fluoro-ruby, dextran tetramethylrhodamine) has been shown to be an ideal long-distance tracer for neuron cells because of its high reliability, sensitivity and good fluorescence intensity characteristics [64]. Re-expression of p75 in the injured motoneurons signified their survival and regeneration [65]. The results of the previous studies provided further support to the idea that p75 plays a beneficial role in neuronal recovery from axonal injury [45, 66]. In our study, it was found that treatment with QCN significantly enhanced the FR-labeled cells and p75 positive motoneurons after avulsion when compared with the vehicle group. In addition, QCN promoted the survival of the motoneurons and accelerated axon regeneration after surgery.

When spinal roots are ripped off from the spinal cord, connection no longer exists between the cell bodies of motoneurons and their axons. Target muscles are deprived of innervation from motoneurons when injury occurs [9, 67]. Long-term denervation results in muscle atrophy, characterized by reduction of muscle weight and muscle fiber size, and higher amounts of fibroblasts [68, 69]. In our study, it was found that the right upper extremities of the vehicle treated-rats exhibited the characteristics as alluded to above. However, treatment with QCN dramatically attenuated the disruption of muscle in a dose-dependent manner, and the related morphological changes in the biceps tissue as a result of avulsion surgery were also markedly remitted by QCN treatment. Trophic support from the regenerated axons facilitates MEP preservation and eases muscular atrophy [70]. After avulsion injury, acceleration of regrowth of axons into the target muscle to take shape the motor endplate is the key to functional recovery. The motor endplate can be used as another important index of nerve regeneration [71]. It was found that the vehicle-treated BPA rats showed smaller and faintly stained MEPs, which was consistent with previous studies [72], while the greater area and clearer appearance of MEPs were seen in the QCN group, resembling those in the normal control group. These results clearly suggested that QCN ameliorated the motor function damage and had a conspicuous protective effect against BPA in rats.

BPA is a damage at the interface of the peripheral and central nervous systems, which creates an inflammatory microenvironment and induces extensive inflammatory factors [73, 74]. Increasing lines of evidence have demonstrated that the activation of microglia cells and astrocytes contributes to the pathophysiology of SCI [75–77]. Activated microglia cells and astrocytes aggravate the inflammatory responses and play detrimental roles in the neuron survival through secreting superabundant pro-inflammatory cytokines, chemokines and neurotoxic factors [78]. In the present study, the QCN-treated rats showed a significant decrease in the activated microglia cells and astrocytes in a dose-dependent manner at week 8 after nerve root avulsion injury. The results indicated that the ameliorative effects of QCN against avulsion injury are closely associated with its anti-inflammatory effects.

On the other hand, root avulsion also causes significant and rapid oxidative stress and the production of reactive oxygen species such as nitric oxide (NO). It has been reported that nNOS, which produces NO through a series of oxidative reactions, can be induced in spinal motoneurons by root avulsion lesion in adult rats, and that the induction of nNOS coincides with the death of the injured motoneurons [5, 65, 73]. Direct evidence from studies shows that application of NOS inhibitors can rescue neuronal death following axonal injury [52, 79, 80]. Besides, Nrf2, a redox-sensitive transcription factor, is involved in cellular protection against oxidative stress through the anti-oxidative responsive element (ARE) to activate the transcription of ARE-regulated phase II antioxidant enzymes, such as HO-1 [81]. HO-1 is essential for the protection of neuronal structure and function. It firms the blood-spinal cord barrier and assuage oxidative stress and white matter damage in the acutely injured murine spinal cord [82]. Studies have also shown that NO donors activates the transcription up-regulation of phase II enzymes, through Nrf2 via ARE in neuronal cells [83], and activation of the Nrf2/HO-1 pathway is critical for neuroprotection [84]. Similarly, our findings revealed that QCN could attenuate the avulsion-induced oxidative injury to prevent motoneuron loss by downregulating the level of nNOS, while upregulating the protein expressions of Nrf2 and HO-1 in the spinal cord.

The precise mechanisms for motoneuron death after axonal injury remain unknown. However, divesting of neurotrophic support is thought to play a crucial role in inducing axotomized spinal cord motoneuron death [41, 85–87]. The family of neurotrophins, including NGF and BDNF, plays an essential role in mediating the survival, differentiation, growth, regeneration, and apoptosis of neurons by binding to respective cell surface receptors and maintaining synaptic connectivity in the adult nervous system [17]. Effects of NGF and BDNF are generally believed to be associated with receptor-linked protein tyrosine phosphorylation, followed by the activation of downstream signal transduction pathways, finally resulting in inhibition of apoptosis [88]. In particular, two intracellular pathways are of importance in mediating survival and/or differentiation of neurons: the Raf/MAPK and the Akt pathways [89]. Under SCI conditions, on the one hand, reduction in the levels of NGF and BDNF and their receptors leads to Akt inhibition and undermines cell survival through the phosphoinositide 3-kinase (PI3-K) pathway [90]. However, in the normal status, Akt may suppress apoptosis and promote neuron survival directly by inhibiting the pro-apoptotic signals Bax, cleaved caspase-9, and cleaved caspase-3 and motivating anti-apoptotic signals Bcl-2 and Bcl-xL [90, 91]. On the other hand, following exposure to neuropathic factors, there is an upregulation in the intracellular signaling activity that is initiated by activated Trk receptors with elevations in the level of B-Raf, p-MEK, and p-ERK, which are proteins central to the MAPK cascades [92]. The present study revealed that QCN treatment significantly alleviated motoneuron apoptosis, at least in part, by activating neurotrophin and Akt signaling pathways, while suppressing MAPK signaling pathway in the avulsion/reimplantation model.

## Conclusions

**Fig. 9:**
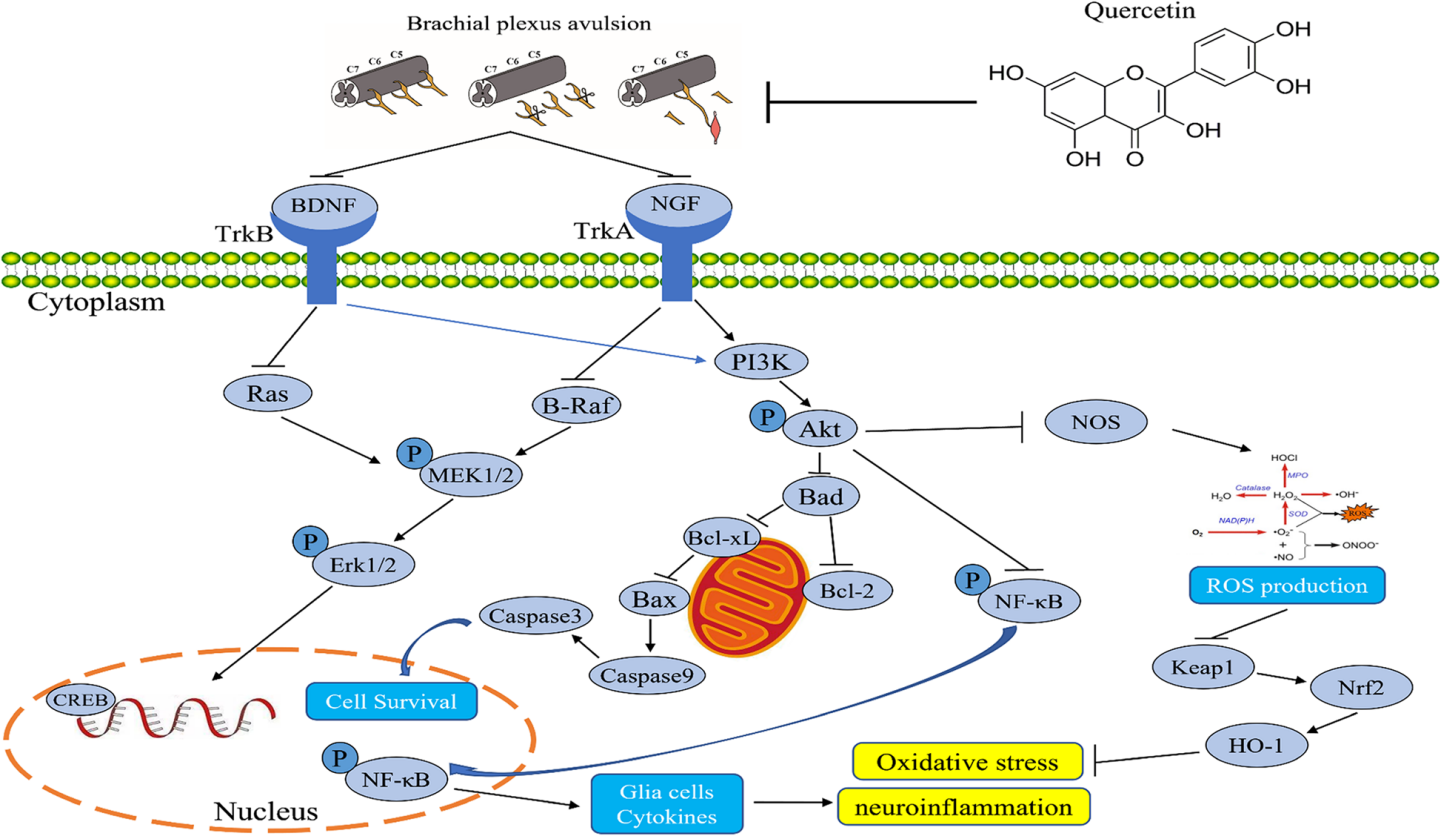
Schematic illustration depicting the putative pathways associated with the therapeutic effect of QCN after BPA in rats. QCN significantly elevated survival and axonal regeneration of motoneurons and enhanced functional recovery after spinal root avulsion/reimplantation in rats. The neuroprotective effects of QCN might be attributed to the inhibition of neuroinflammatory response and oxidative stress, partially via activating neurotrophins, such as NGF and BDNF, and modulating the Akt and MAPK signaling pathways

In summary, this study for the first time demonstrated that QCN significantly elevated survival and axonal regeneration of motoneurons and enhanced functional recovery after spinal root avulsion/reimplantation in a rat model. The neuroprotective effects of QCN are attributed to the inhibition of neuro-inflammatory response and oxidative stress, partially via activating neurotrophins, such as NGF and BDNF, and Akt signaling pathway, while attenuating MAPK pathway (Fig. 9). Taken together, QCN is a promising therapeutic option to assist reimplantation surgery in the treatment of BPA. Further in-depth studies are warranted to fully illuminate the precise cellular and molecular mechanisms underlying the protective effects of QCN on motoneurons after root avulsion injury.

## Supplementary Information

**Additional file 1.** 

## Data Availability

The data that support the findings of this study are available from the corresponding authors upon reasonable request.

## Abbreviations

α-BTX: α-Bungarotoxin
AChR: Acetylcholine receptor
ARE: Anti-oxidative responsive element
Bax: B cell lymphoma-2-associated X
Bcl-2: B cell lymphoma-2
Bcl-XL: B cell lymphoma-extra large
BDNF: Brain-derived neurotrophic factor
BPA: Brachial plexus avulsion
ChAT: Choline acetyltransferase
CNS: Central nervous system
FR: Fluoro-Ruby
GFAPGlial: Glial fibrillary acidic protein
HO-1: Heme oxygenase-1
Iba1: Ionized calcium-binding adaptor molecule 1
MAPK: Mitogen-activated protein kinase
MEP: Motor endplate
NGF: Nerve growth factor
nNOS: Neuronal nitric oxide synthases
p75: Low-affinity nerve growth factor receptor
Nrf2: Nuclear factor-erythroid 2-related factor 2
p-ERK: Phospho-extracellular signal-regulated kinase
PFA: Paraformaldehyde
PI3-K: Phosphoinositide 3-kinase
p-MEK: Phospho-MAKP/ERK kinase
PNS: Peripheral nervous system
QCN: Quercetin
ROS: Reactive oxygen species
SCI: Spinal cord injury
TGT: Terzis grooming test
Trk: Tropomyosin-related kinase
