# Supplementary material for: Quercetin enhances survival and axonal regeneration of motoneurons after spinal root avulsion and reimplantation: experiments in a rat model of brachial plexus avulsion
Source: Inflamm Regen. 2022 Dec 1;42:56. doi: 10.1186/s41232-022-00245-3 (PMC9714227; doi:10.1186/s41232-022-00245-3)

**Supplementary data**

Fig. 6C

Nrf-2

1
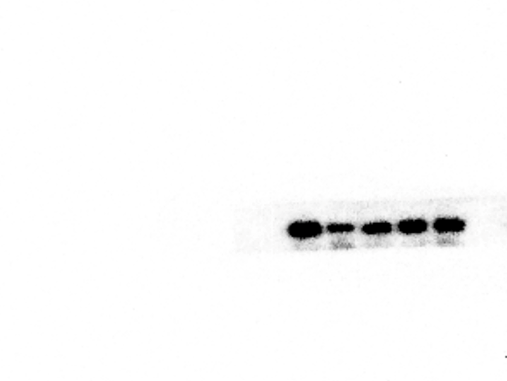


2
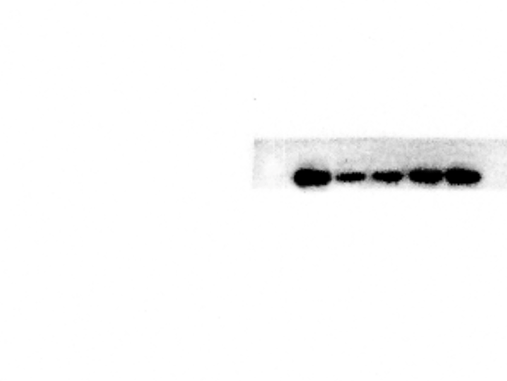


3
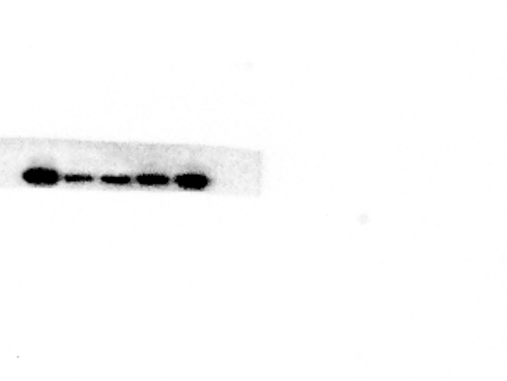


HO-1

1
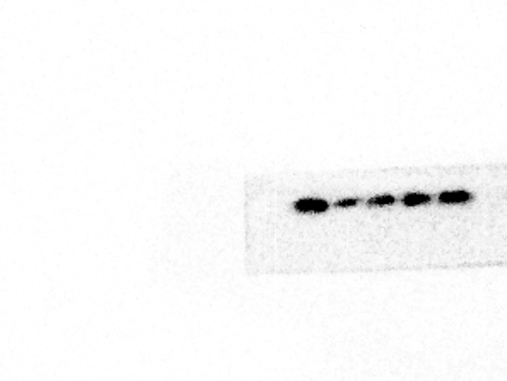


2
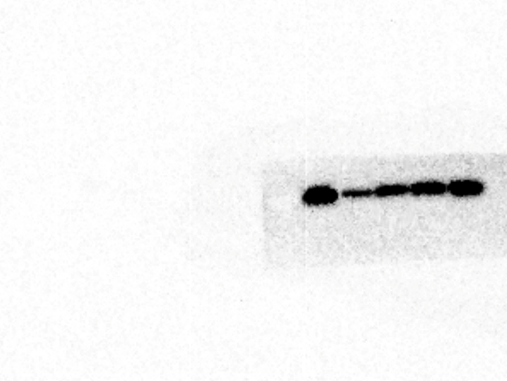


3
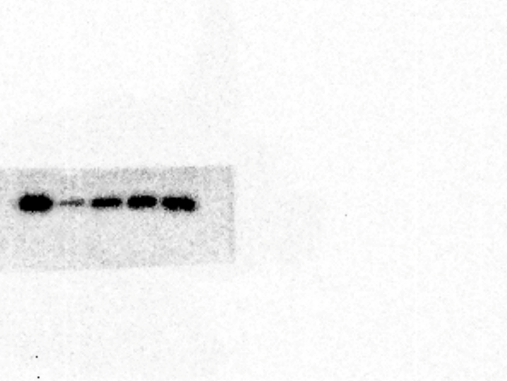


GAPDH

1
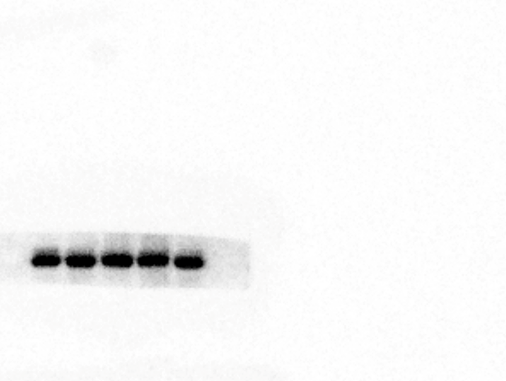


2
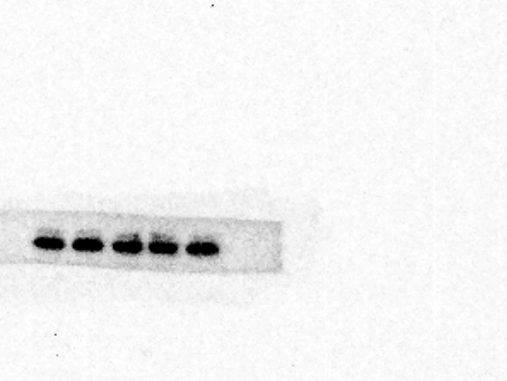


3
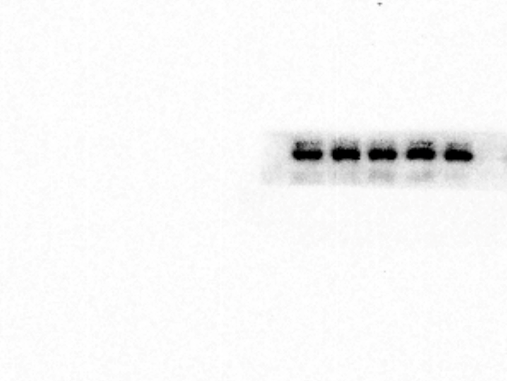


Fig. 7A

NGF

1
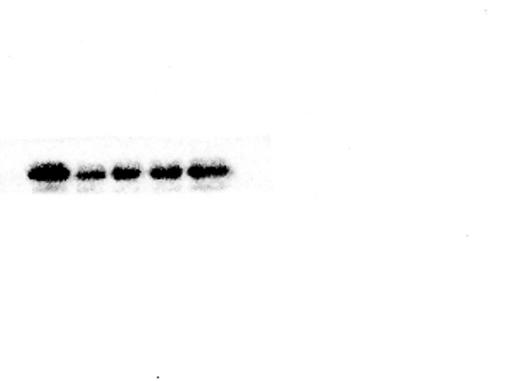


2
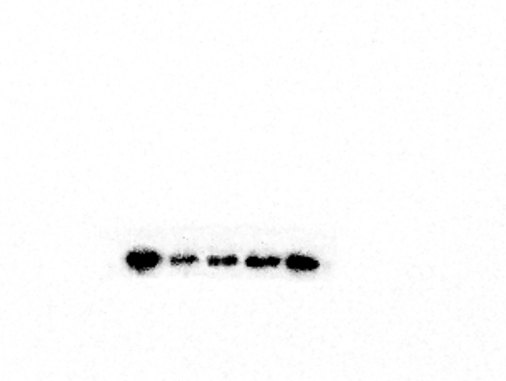


3
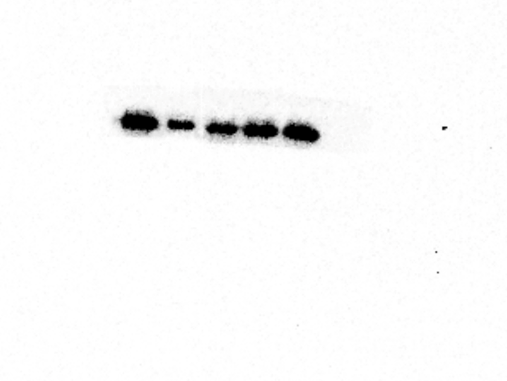


BDNF

1
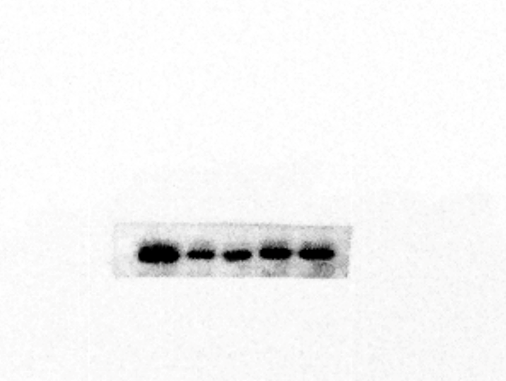


2
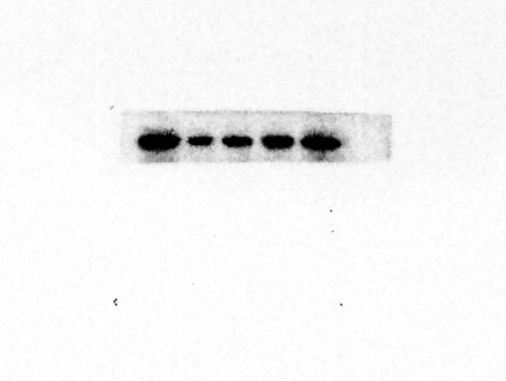


3
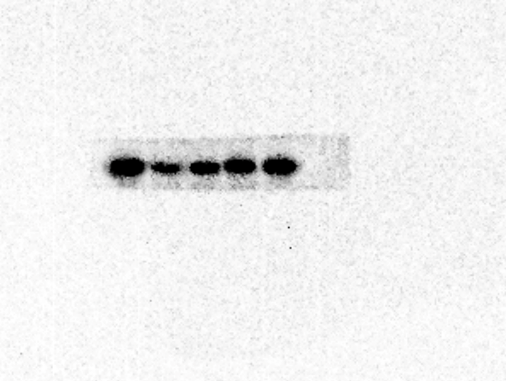


Fig. 7D

p-Akt

1
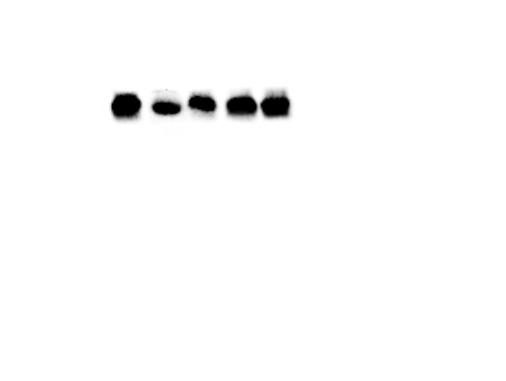


2
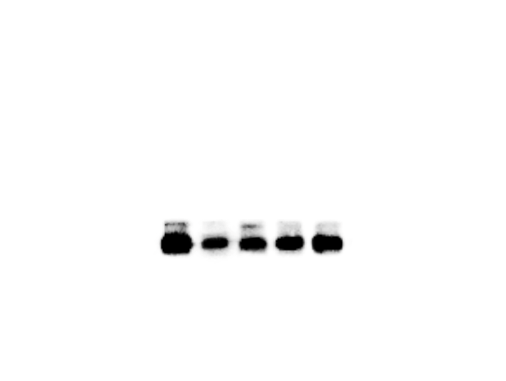


3
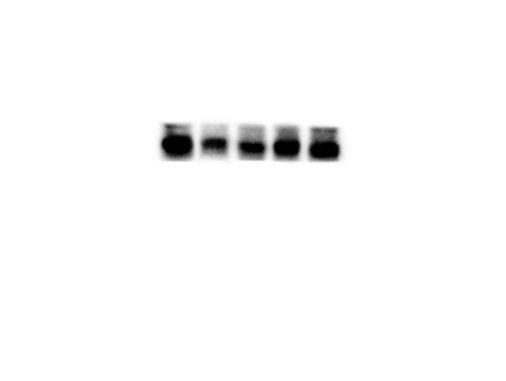


Akt

1
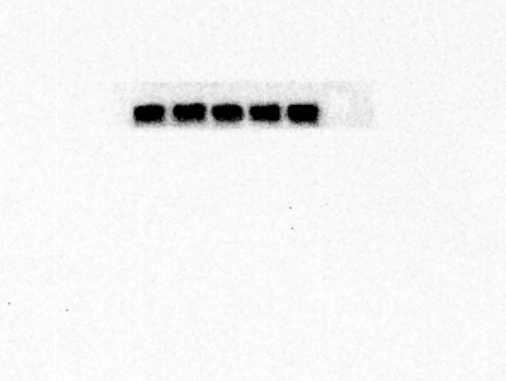


2
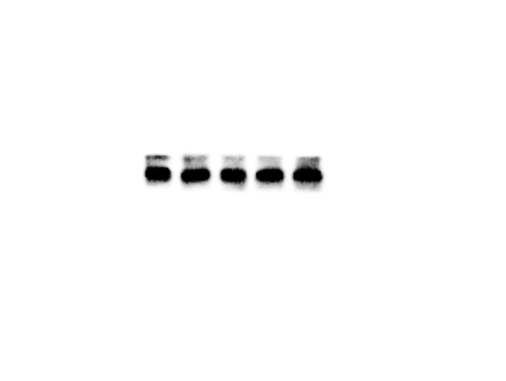


3
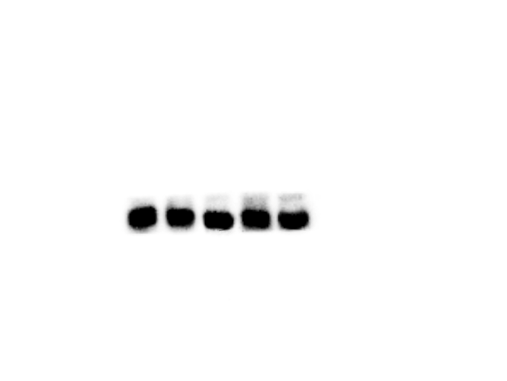


Bcl-2

1
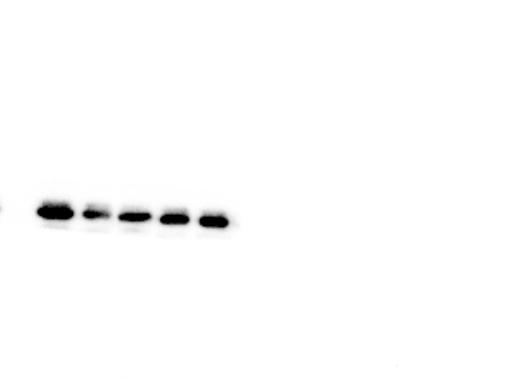


2
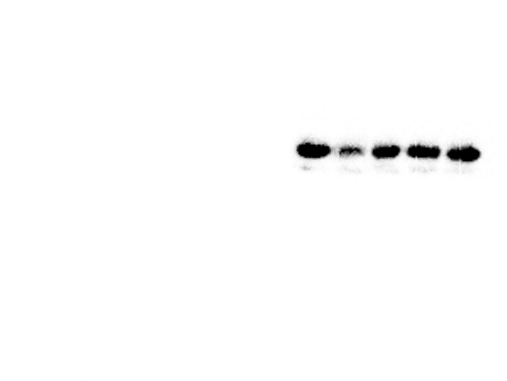


3
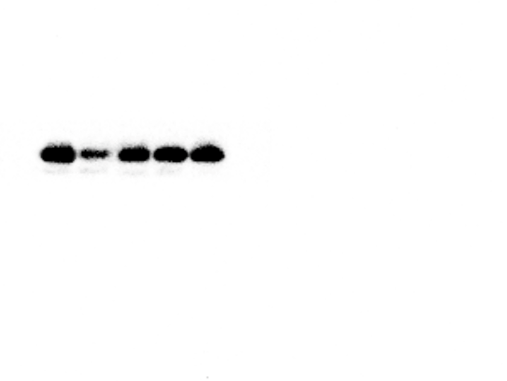


Bcl-xL

1
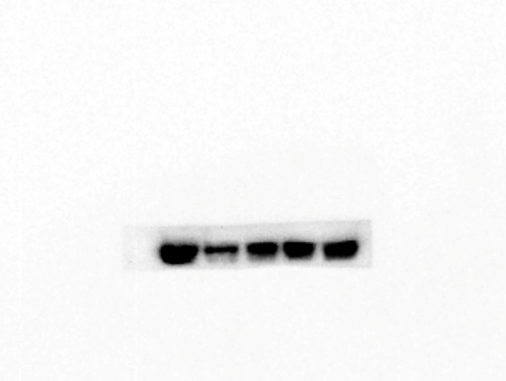


2
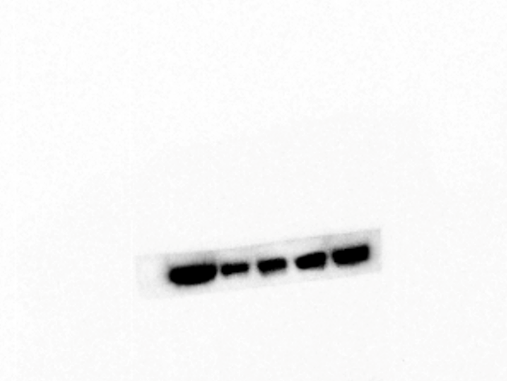


3
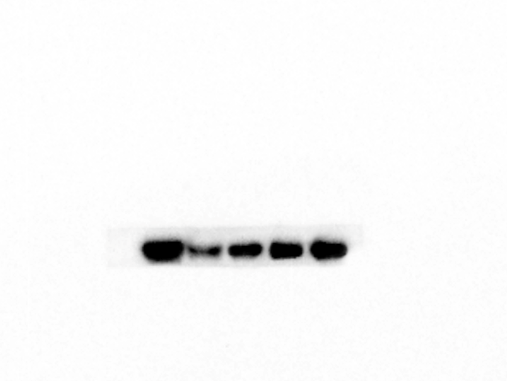


Bax

1
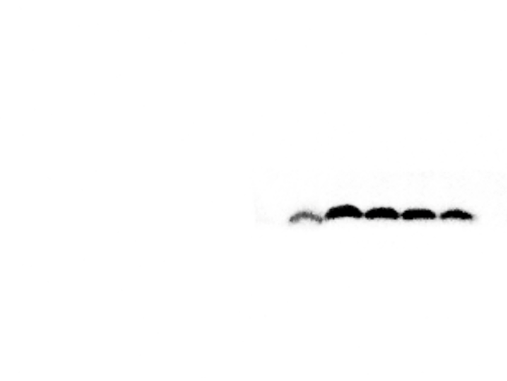


2
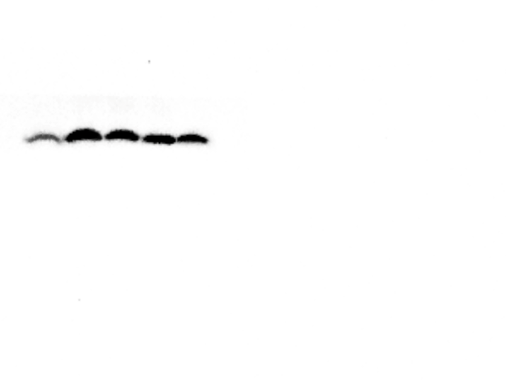


3
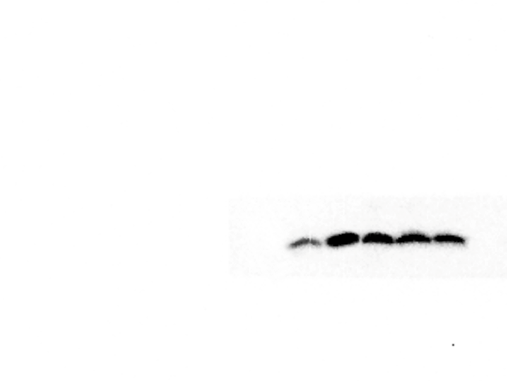


Cleaved Caspase 9

1
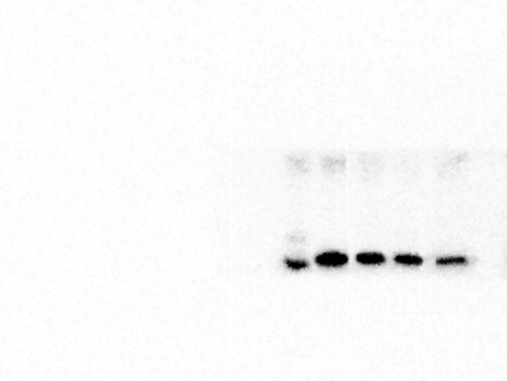


2
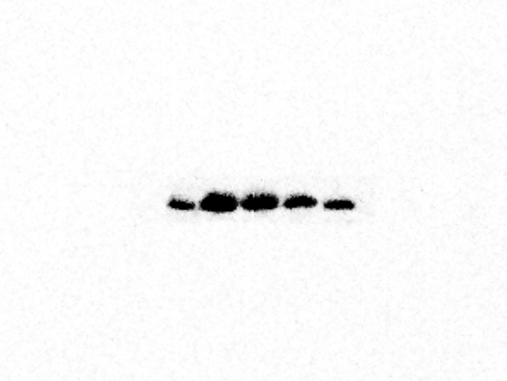


3
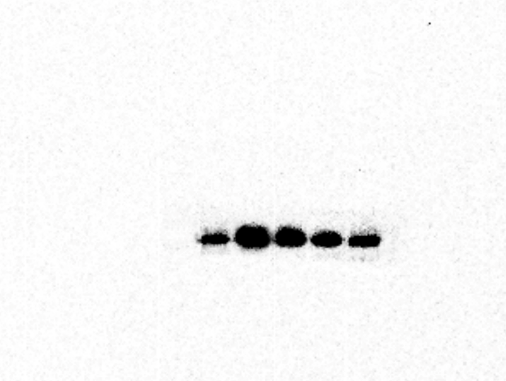


Cleaved Caspase 3

1
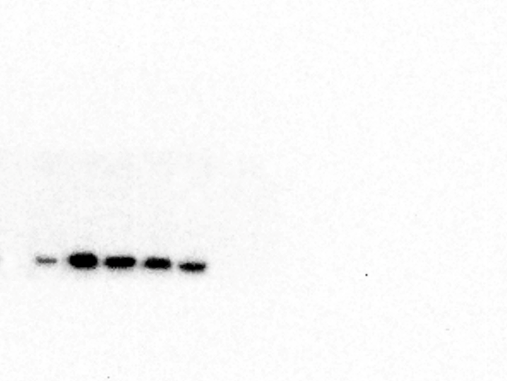


2
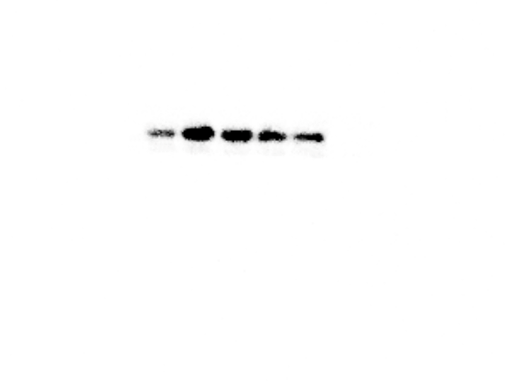


3
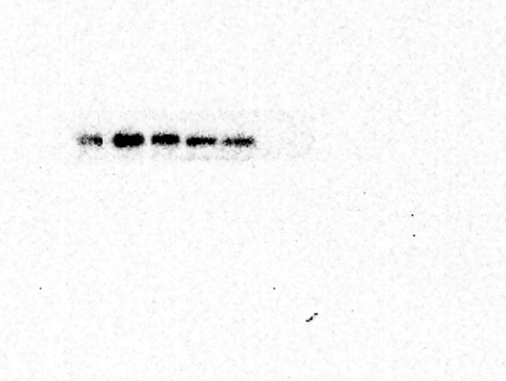


Fig. 8A

B-Raf

1
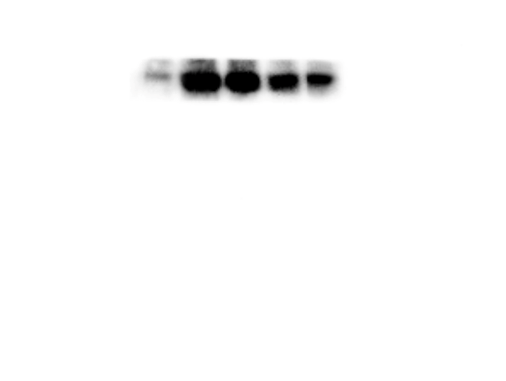


2
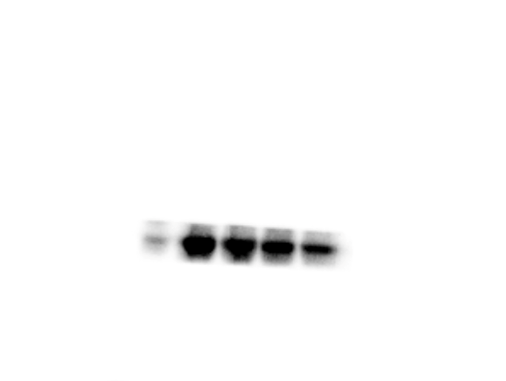


3
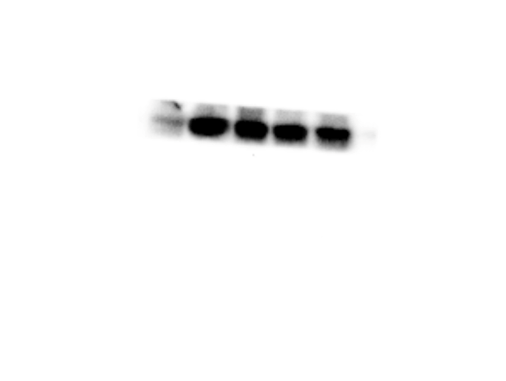


p-MEK

1
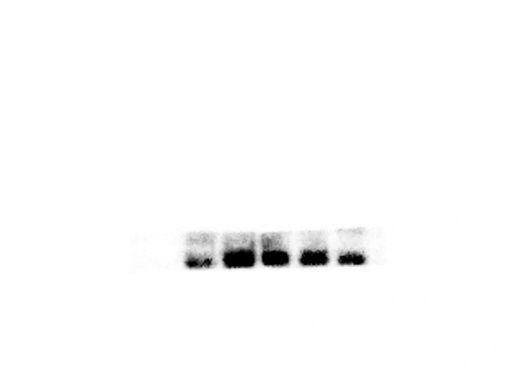


2
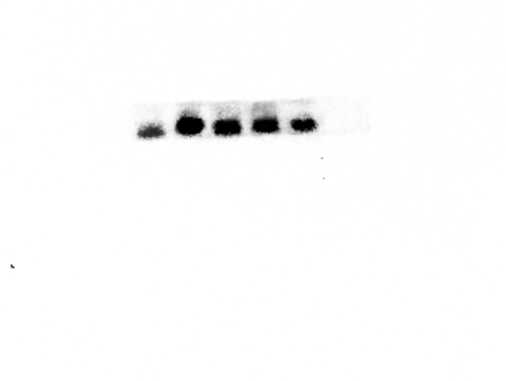


3
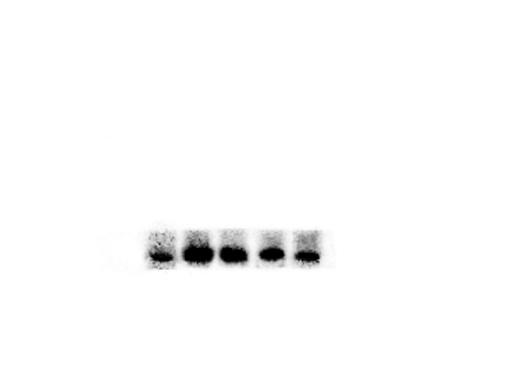


MEK

1
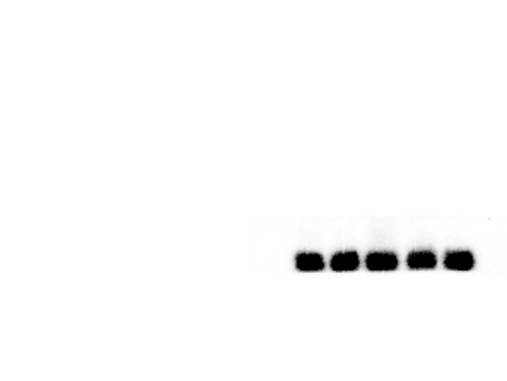


2
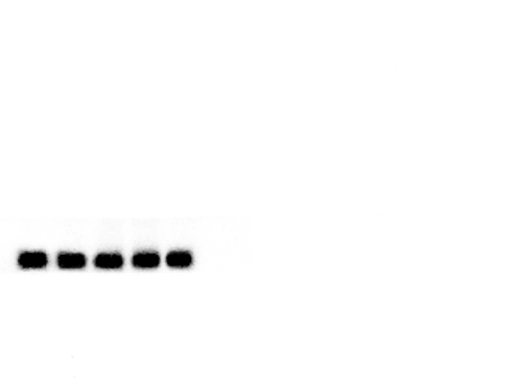


3
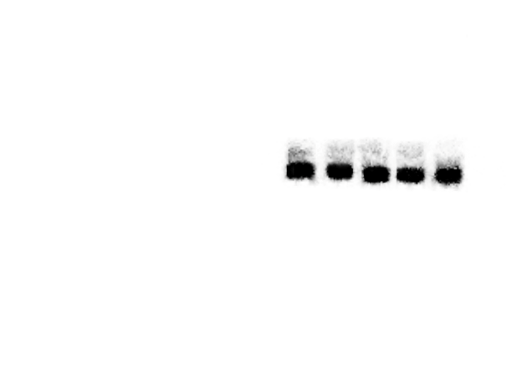


p-ERK

1
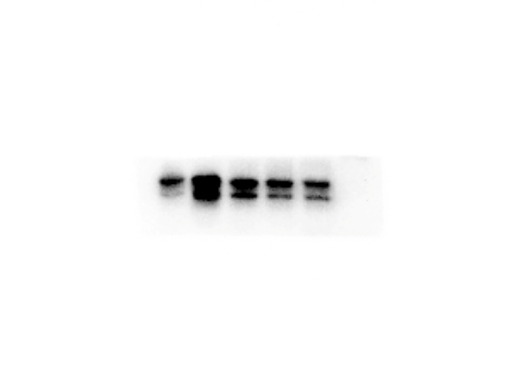


2
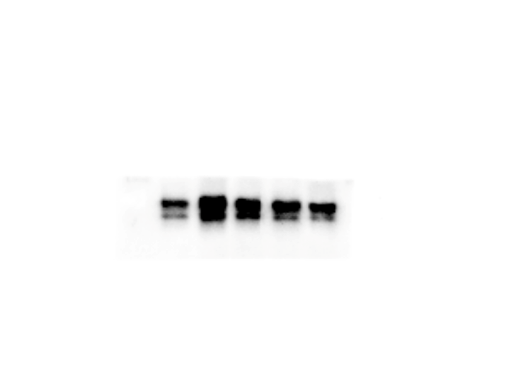


3
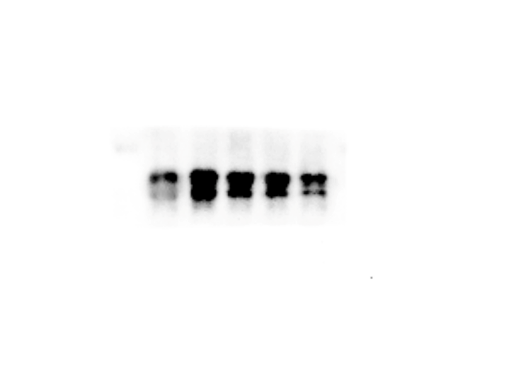


ERK

1
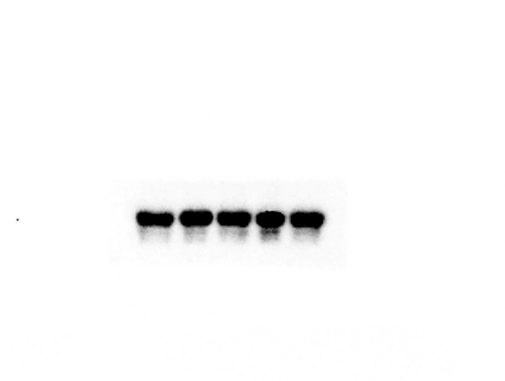


2
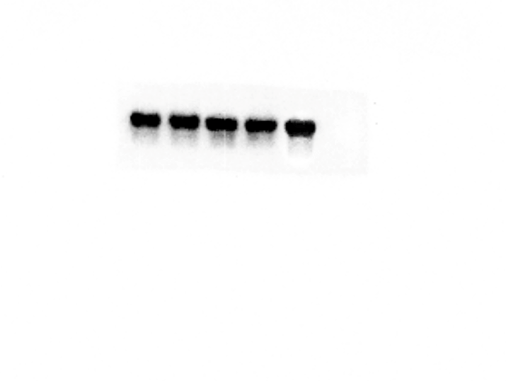


3
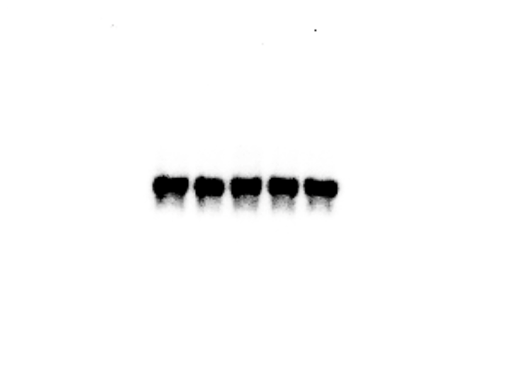


Fig. 7A & D and Fig. 8A

GAPDH

1
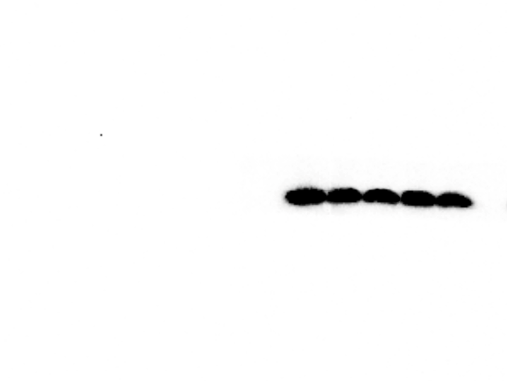


2
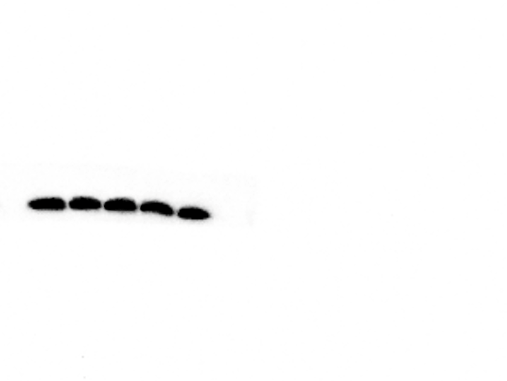


3
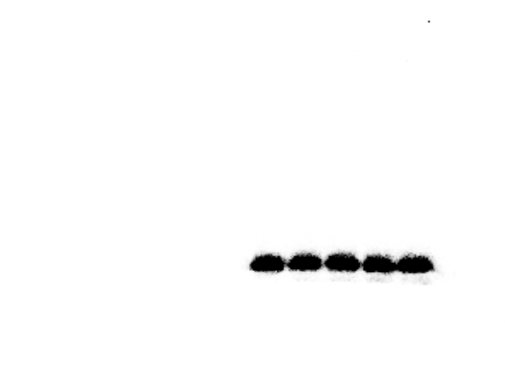

Supplement: Supplementary file 1 — Additional file 1. [file 41232_2022_245_MOESM1_ESM.docx]
